# Hif-1 responsive IFFLs to explain specific transcriptional responses to cycling hypoxia in cancers

**DOI:** 10.1038/s41540-025-00612-z

**Published:** 2025-11-24

**Authors:** Xihua Qiu, Yamin Liu, Paola Vera-Licona, Eran Agmon, Kshitiz, Yasir Suhail

**Affiliations:** 1 Department of Biomedical Engineering, Univeristy of Connecticut, Storrs, CT USA; 2 Department of Biomedical Engineering, University of Connecticut Health, Farmington, CT USA; 3 Center for Cell Analysis and Modeling, University of Connecticut Health, Farmington, USA

**Keywords:** Cancer, Genetic interaction

## Abstract

The adaptive response of cancer cells to hypoxia, a key microenvironmental factor in solid tumors, is orchestrated by Hypoxia-inducible factor 1 (HIF-1). Recent evidence indicate that oxygen tension in tumor is dynamic, with hypoxia being frequently unstable, or cycling. Cycling hypoxia is associated with specific phenotypic outcomes for the cancers. Transcriptomic analysis shows that for most genes, expression changes in cycling hypoxia lie expectedly in between the change caused by stable hypoxia, suggesting multi-cycle averaging of dosage in the oxygen tension, and likely HIF-1 induced transcription. However, a small subset of genes show an oscillation/cycling hypoxia specific response, suggesting that the transcriptional machinery of these genes may interpret cycling HIF-1 activity differently from stably high HIF-1 activity. Here, we model a gene regulatory circuit, the incoherent feed-forward loops (IFFLs) to illustrate that there are parameter regimes in such genetic circuits where oscillatory specific transcription is plausible. In these IFFL models, HIF-1 regulates gene transcription of a target gene directly, as well indirectly via another transcription factor with an opposite effect on gene transcription. This IFFL circuit is able to generate gene expression of certain target genes that is more extreme than either normoxia or stable hypoxia, and this nonlinear IFFL behavior can result from either the dynamic nature or even the intermediate, time averaged hypoxic signal Supplementary Information 1 (*Steady state analysis of IFFL circuits*). This gene circuit also allows us to search for plausible signaling intermediaries involved in the IFFL mediated cycling hypoxic response. Finally, we present experimental evidence suggesting that HIF-1 can form IFFLs with two key transcription factors p53, and Notch1, resulting in cycling hypoxia-specific gene expression linked to breast cancer progression and poor prognosis. Our work aims to draw attention to genetic circuits as plausible mechanisms where temporal fluctuations in the tumor microenvironment may directly inform downstream transcription. These ideas could identify hitherto unknown HIF-1 driven mechanism of cancer progression contributing to emergent tumor heterogeneity.

## Introduction

As tumors grow, they frequently outstrip their supply of oxygen, leading to the development of hypoxic regions. Hypoxia is a key microenvironmental factor in solid cancers, influencing many aspects of cancer growth, metastasis, and drug resistance^1,2^. The cellular response to hypoxia is regulated by HIF-1 (Hypoxia-inducible factor 1), a transcription factor which acts as the master regulator of cellular responses to low oxygen levels (hypoxia)^3,4^. HIF-1 is a heterodimer, composed of the oxygen tension regulated HIF-1-alpha, and constitutively expressed HIF-1-beta. HIF-1 is stabilized in low oxygen conditions, and acts as a key transcription factor (TF), regulating the expression of numerous genes involved in angiogenesis, metabolism, and cell survival to adapt to oxygen deprivation^5^. Consequently, the advent of hypoxia can have a profound effect on nearly all steps in the metastatic cascade^6^.

In the recent decade, development of in vivo imaging technologies have revealed that hypoxia is frequently not temporally stable, leading to fluctuations or cycling of oxygen tension^7–9^. Our own works, as well as others, have shown that cycling, or oscillatory, hypoxia can result in gene expression patterns associated with low patient survival, altered tumor inflammation, and stromal activation^7,10–13^. Furthermore, we had shown that the stability of HIF-1 could vary over time due to accumulated lactate in the tumor milieu, which facilitates non-canonical degradation of HIF-1 via chaperone-mediated autophagy^14,15^. Crucially, while HIF-1 is known to suppress cell proliferation both transcriptionally by elevating tumor suppressor p21 and p27^16^, or counteracting the oncogenic effect of Myc^17^, as well as non-transcriptional mechanisms^18^, oscillatory/cycling hypoxia allowed the cells to continue to proliferate^10,14^, partially addressing the conundrum why tumors continue to proliferate in hypoxia.

Most of the effects of cycling hypoxia previously reported, as well as our own reported findings, indicate that the downstream HIF-1 driven gene expression is averaged for cycling hypoxia in between normoxia and stable hypoxia. A plausible explanation is that cycling hypoxia elicits a “sub-hypoxic” transcriptional response averaged over multiple cycles. This sub-hypoxic response, wherein the gene expression in cycling hypoxia is in between that of normoxia and stable hypoxia explains many of the observed phenomena, including increased cell proliferation, respiration etc.

However, gene expression analysis of HeLa^14^, MDA-MB-231^10^, as well as single cell RNAseq analysis of a population of various cell types in breast cancer tumor microenvironment showed many genes that had cycling/oscillatory hypoxia-specific gene expression^19^. These genes unexpectedly show either a directionally congruent but more extreme response to stable hypoxia, or even opposite to stable hypoxia. Many of these genes could have a profound effect on cancer progression, and considering that they are induced specifically by cycling hypoxia, may provide hitherto unknown HIF-1 driven mechanisms of cancer progression.

How could a gene expression be driven specifically by cycling hypoxia, more than, or oppositely to stable hypoxia? Here, we model these patterns using Incoherent Feed Forward Loop (IFFL) motifs in the gene transcriptional machinery involving HIF-1 as the master transcriptional regulator^20^. IFFLs have been previously shown to be a strongly selected motif in prokaryotic transcriptional network^20^, and can differentiate variable signal from stable signal^21^. As HIF-1 is a master TF, regulating the expression of many other TFs, which in turn regulate downstream gene expression, IFFLs could easily form where HIF-1 and the TF induced by HIF-1 together regulate a target gene.

We analytically modeled various IFFL types, which could be formed between HIF-1, and a target gene with incorporation of another HIF-1-driven TF. Mathematical modeling revealed plausible regimes where cycling/oscillatory HIF-1, or even intermediate hypoxic signal specific Supplementary Information 1*(Steady state analysis of IFFL circuits)* gene expression patterns could arise. Surprisingly, we found that a single IFFL subtype could give rise to many different cycling/oscillatory hypoxia-specific gene expression patterns that we identified using triple-negative breast cancer MDA-MB-231 cells.

Finally, we found two key regulators, which could potentially act as candidate TFs to create IFFLs with HIF-1, the tumor suppressor p53, and Notch1, regulating expression of several oscillation-specific genes. Measuring gene silencing for TP53 and NOTCH1, and measuring RNA levels by RNAseq in normoxia, stable hypoxia, and cycling hypoxia in MDA-MB-231 cells, we found many genes which form an IFFL gene regulatory circuit with HIF-1 along with either p53, or Notch1. Our mathematical model not only provides a mechanistic understanding of how gene expression patterns could emerge specifically in response to cycling hypoxia, but also demonstrate that microenvironmental dynamics can translate into specific transcriptomic, and therefore phenotypic responses in cancers.

## Results

### Gene expression in triple-negative breast cancer cells exhibit cycling hypoxia-specific patterns

**Fig. 1:**
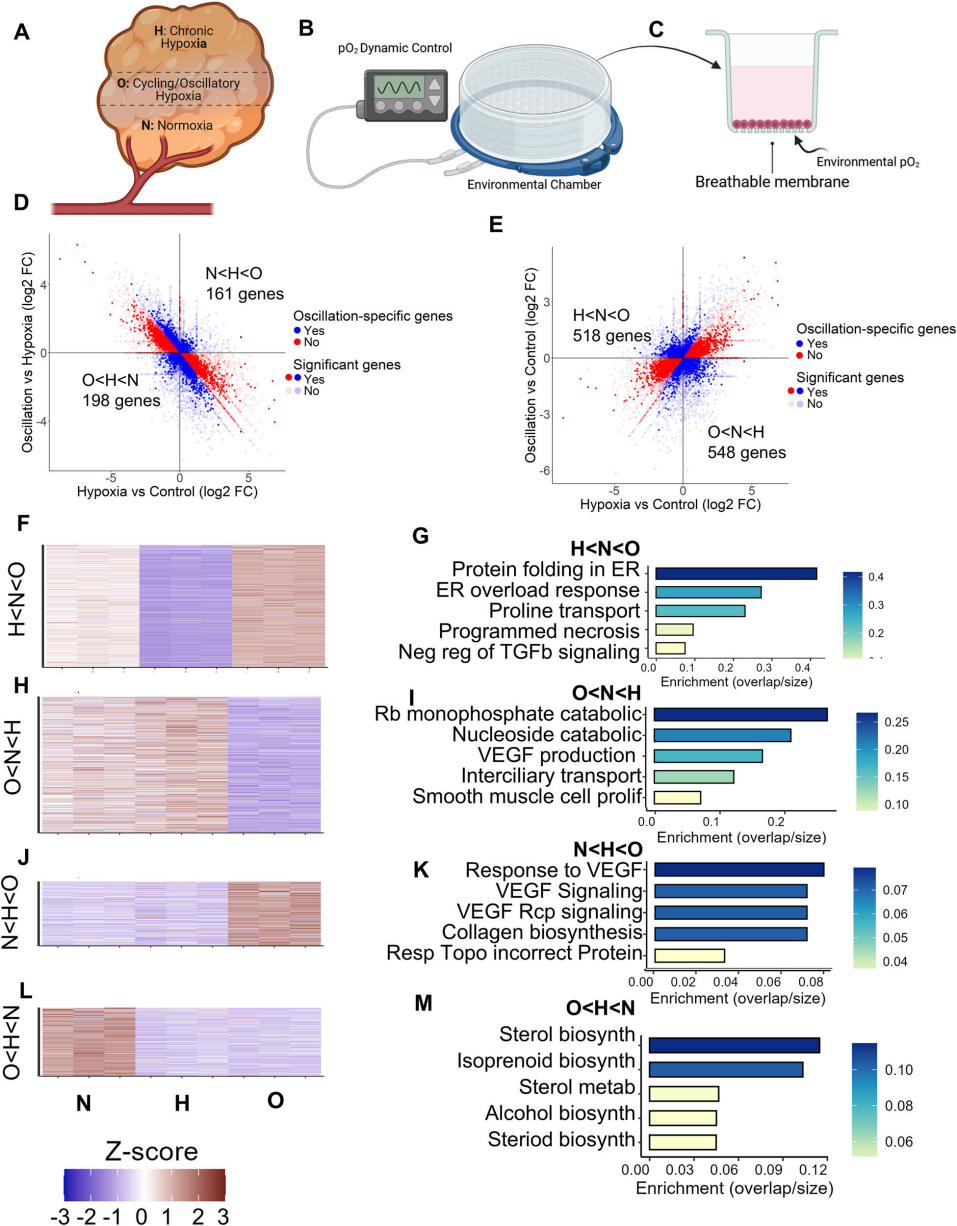
Cycling/oscillatory hypoxia results in a specific pattern of gene expression in MDA-MB-231, distinct from stable/chronic hypoxia. **A** Schematic showing a sub-optimally vascularized solid tumor with regions of chronic hypoxia, cycling hypoxia, and normoxia in a tumor due to the spatial distribution of vasculature, created using BioRender. **B**, **C** Experiment setup to control hypoxic dynamics in cell culture. **D** Scatter plot showing relative differential expression (in log2 fold change) between O vs H (y-axis), and H vs N (x-axis); Quadrants show gene expression following the oscillation specific pattern (H < N < O) top left, (O < N < H) bottom right. **E** Scatter plot showing relative differential expression (in log2 fold change) between O vs N (y-axis), and H vs N (x-axis); Quadrants show oscillatory specific patterns (N < H < O) bottom left, and (O < H < N), top right. **F**–**M** Heatmaps (left column) and top enriched pathways (right column), for genes with oscillation specific patterns H < N < O, O < N < H, N < H < O, and O < H < N respectively.

To ascertain the extent of gene expression changes in response to stable and dynamic oxygen tension, in triple negative breast cancer cells (TNBCs), we used MDA-MB-231 cells and treated them with normoxia (N), stable hypoxia (H), and oscillatory/cycling hypoxia (O) for 48 h (Fig. 1A). TNBCs frequently develop hypoxic cores, with peripheral regions perfused sub-optimally resulting in fluctuating or cycling hypoxia (Fig. 1A)^7,9^. As molecular oxygen has extremely low solubility in aqueous solutions^22^, dynamic oxygen stimulation is very difficult to control precisely as experienced by the cells, with soluble pO_2_ being highly sensitive to height of medium, air pressure, and many parameters. We therefore cultured MDA-MB-231 cells on gas-permeable surfaces^10^, exposed from the bottom to the ambient oxygen, so that any change in the ambient pO_2_ is directly sensed by the cultured cells from the bottom of the plate.

Cultured cells were kept in a sealed chamber connected to a dynamic gas mixing controller, and subjected to normoxia (N: atmospheric O2, ~21%), hypoxia (H: 1% O_2_), and cycling/oscillatory hypoxia (O) in between N, and H with a period of 90 min for a total of 48 h (Fig. 1B, C). Cells were immediately lysed, RNA collected, and sequenced. Differential gene expression analysis revealed that hypoxia expectedly had a dramatic effect on gene expression vs normoxia. With the adjusted p-values set for significance of 0.05 for all three fold changes, we counted the number of significant oscillation -specific genes in different quadrants (Fig. 1D, E). As expected from our default hypothesis, for most genes, cycling hypoxia resulted in an averaged response between N and H for most of the genes (Fig. 1D, E). Surprisingly, we also found a large number of genes with cycling hypoxia specific changes in expression (Fig. 1D, E).

We categorized the patterns of cycling hypoxia-specific gene expression in 4 broad categories defined by the significant differences between the three conditions arranged in terms of their transcripts per million (TPM) values. The first two patterns, O < H < N, and N < H < O consist of genes whose expression in cycling hypoxia was more extreme than in stable hypoxia (Fig. 1D), while the other category included H < N < O, and O < N < H genes where cycling/oscillatory hypoxia had an opposite effect than stable hypoxia (Fig. 1E). In none of the cases is the gene expression pattern explainable by the hypothesis that cycling hypoxia results in a sub-hypoxic, averaged response between normoxic and hypoxic states.

Gene set enrichment analysis of O genes revealed some phenotypic coherence. The 518 genes which showed a downregulation in stable hypoxia, but increased in cycling hypoxia (Fig. 1F) were enriched in Protein folding response (Fig. 1G), as we have previously reported, and shown to be linked to low patient survival^10^. Gene Ontologies (GO) enriched in 548 genes which were decreased in cycling hypoxia, oppositely to stable hypoxia were of variable types, including ribosomal and nucleoside catabolic processes, as well as VEGF production (Fig. 1H, I). Interestingly, genes that responded to cycling hypoxia in the same direction as stable hypoxia, but with a more pronounced change in expression, were enriched for GO terms related to angiogenesis and collagen biosynthesis (Fig. 1J, K). Cycling hypoxia resulted in decreased expression of genes associated with various biosynthetic pathways (Fig. 1L,M), similar to stable hypoxia, although directionally more extreme. Overall, our data with MDA-MB-231 cells show a large number of genes which had a specific effect on expression by cycling/oscillatory hypoxia vs stable hypoxia, suggesting that an indirect regulatory architecture, such as an incoherent feed-forward loop (IFFL), may interpret fluctuating signals differently from stable ones.

### Incoherent Feed Forward Loops (IFFL) driven by HIF-1 as plausible regulatory units for cycling hypoxia-specific gene expression

We sought to understand how do so many genes specifically respond to cycling hypoxia, even oppositely to stable hypoxia. Considering that gene expression regulatory networks usually feature high interconnectedness and complexity, many genes are regulated through complex networks of transcription factors^23^. These networks include positive and negative feedback loops, among other sophisticated regulatory mechanisms^24^. Based on this information, we might speculate that a significant proportion of human genes could be modeled using differential equations involving transcription factor regulation^25^. HIF-1 is a master transcriptional regulator, transcribing a large number of genes, many of which code for transcription factors^26^, creating a potential for formation of multi-protein regulatory circuits.

Among the many other regulatory mechanisms, incoherent feedforward loops (IFFLs) have been mathematically described as being capable of discriminating between variable and stable signals^27,28^. Furthermore, IFFLs have been shown to be evolutionarily selected motifs in transcriptional regulatory networks in many species^28^. We therefore asked if IFFLs with HIF-1 being the driver TF may explain some of these patterns.

**Fig. 2:**
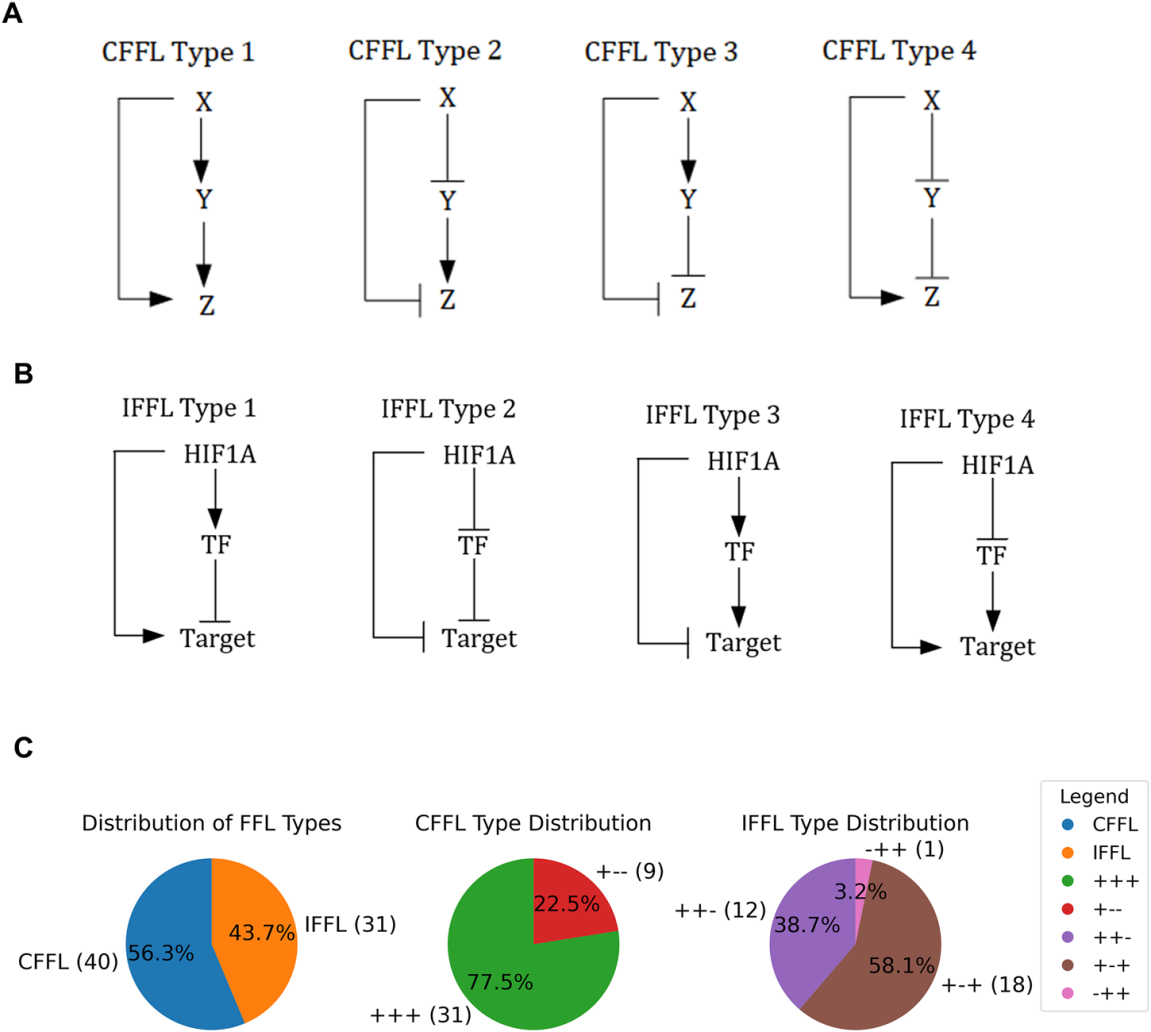
Gene regulatory feed forward loops involving activating and inhibitory interactions. **A** All possible coherent feed forward loops. The overall effect of driving gene X on the target gene Z is in the same direction from both the direct interaction (X->Z) and the indirect interaction (X - > Y - > Z). **B** All possible incoherent feedforward loops, where the direct and indirect effects of X on Z are in opposite regulatory directions (activating and inhibitory). **C** Relative distribution of FFLs, both coherent (CFFLs) and incoherent (IFFLs) identified from literature mined through Ingenuity Pathway Analysis.

Feed-forward loops (FFLs) are a general class of motifs composed of 3 elements, wherein a TF X that regulates a second TF, Y, and both X and Y regulate the target gene Z^20^. FFLs could be coherent, or incoherent, based respectively on the congruency, or incongruency of the parallel arm connecting the regulating TF X to the target Z (Fig. 2A-B). IFFLs have been previously shown to be able to discriminate between variable and stable input signal X^27,29^. The IFFL consists of two parallel paths—direct and indirect—from X to Z, which are functionally opposite: one is activating, and the other is repressive. The direct path consists of a single directed edge, and the indirect path is a cascade of two directed edges, such that their product of activating sign is opposite to the direct edge. It is to be noted that IFFLs unlike FFLs, are highly selected in evolution, and are considered as a gene regulatory motif^20,27^. There are 4 potential IFFLs (Fig. 2B).

We analytically modeled each of them to test the possibility of obtaining cycling/oscillatory hypoxia (O) specific gene expression patterns reminiscent of the data collected in MDA-MB-231 cells in N, H, and O conditions. Using curated transcriptional regulatory relationships from the TRRUST database and Ingenuity Pathway Analysis (IPA), we identified 31 incoherent feedforward loops (IFFLs) and 40 coherent feedforward loops (CFFLs) in which HIF-1 functions as the master regulator (Fig. 2C, Supplementary Data 1). Among them, the majority of the CFFLs (77.5%) belong to CFFL type 1 (+ + +), while the remaining 22.5% fall under CFFL type 3 (+ − −). Similarly, for the IFFLs, type 1 (+ − +) accounts for 58.1%, followed by type 3 (+ + −) at 38.7%, and a small proportion (3.2%) is classified as type 4 (−++).

### Mathematical modeling of IFFLs

**Fig. 3:**
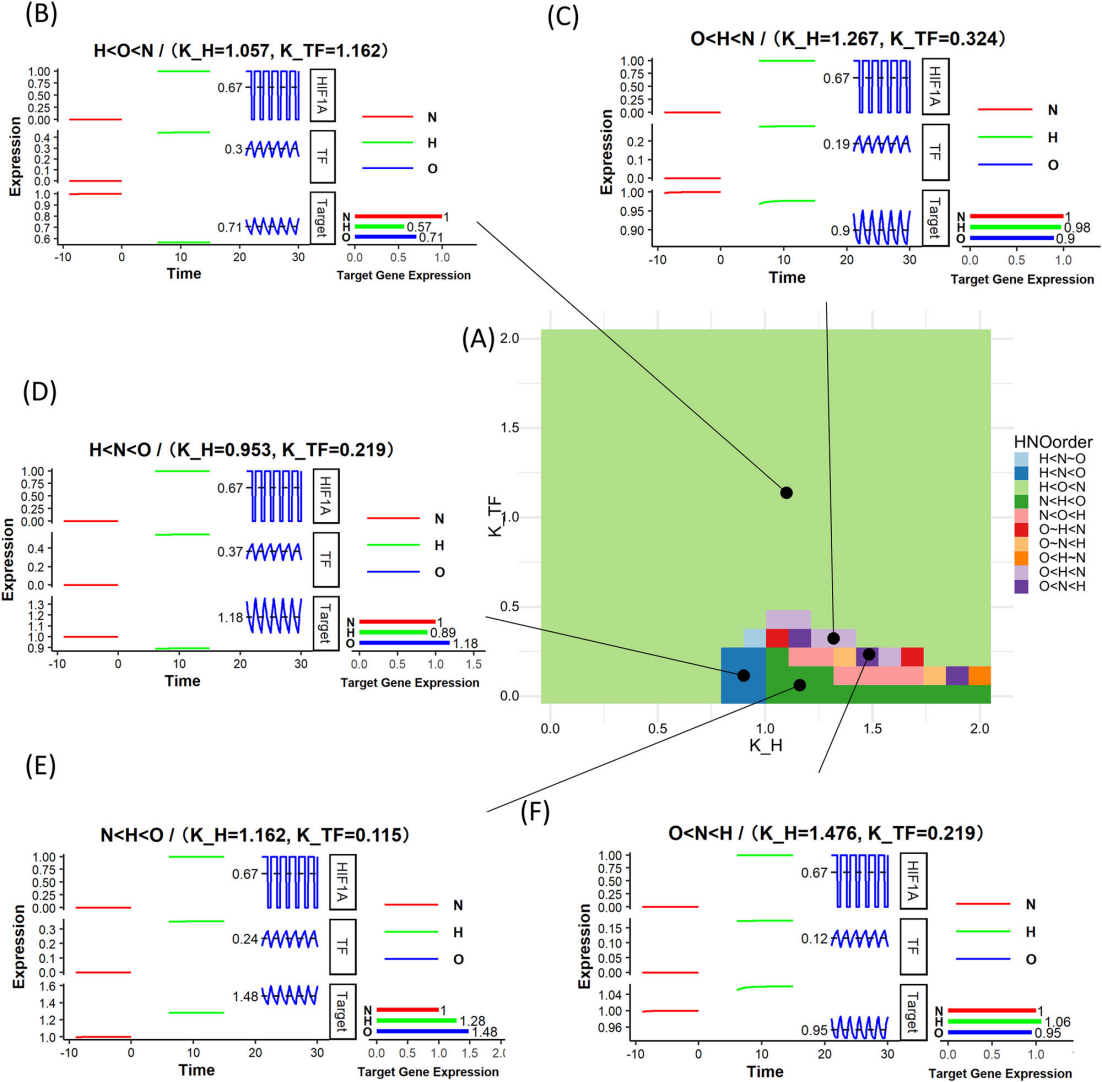
IFFLs with multiplicative effect of HIF-1 and HIF-1 activated TF on target gene expression show cycling/oscillatory hypoxia specific patterns. Phase diagram and example dynamics of IFFL Type 3 circuit with multiplicative inhibition (Eqs. 1 and 2). **A** Phase diagram showing the relative order of the target gene expression in Normoxia (N), Hypoxia (H), and Oscillatory Hypoxia (O). **B**–**F** Example dynamics of the driving HIF1A that is set at 1 for hypoxia, 0 for normoxia, and pulsating 1 h of hypoxia and half an hour of normoxia for oscillatory hypoxia, the intermediate transcription factor, and the Target gene. All gene expression values are in arbitrary units. H, N, O order pattern given only in terms of the target gene expression.

We mathematically modeled all four types of IFFLs using ordinary differential equations. As an example, we show simulated results of the type 3 IFFL (schematic in Fig. 2B) gene circuit driven by HIF-1 in Fig. 3, a regulatory module where HIF-1 activates a secondary transcription factor (TF), which, in turn, regulates a downstream target gene. Simultaneously, HIF-1 directly downregulates the same target gene, creating a balance between activation and inhibition. For nonlinear interactions at each regulatory edge, this circuit shows regimes wherein the target gene expression is driven selectively by cycling hypoxia. Equation (1) describes the expression of the intermediary transcription factor (TF) being driven by HIF-1 binding on its enhancer region, with the nominal production rate of ${\beta }_{{TF}}$, and degradation rate of ${\alpha }_{{TF}}$ that is linear with the amount of TF present. Equation (2) describes the time evolution of the target gene expression level, driven by TF binding on its enhancer region and HIF-1 binding on its silencer region.

1 $$\frac{d\text{TF}}{{dt}}=\frac{{\left(\frac{{HIF}}{{K}_{H}}\right)}^{N}}{{\left(\frac{{HIF}}{{K}_{H}}\right)}^{N}+1}\cdot {\beta }_{{TF}}-{\alpha }_{{TF}}\cdot {TF}$$

2 $$\frac{{dTarget}}{{dt}}={\beta }_{{Target}}\left(\frac{{\left(\frac{{TF}}{{K}_{{TF}}}\right)}^{N}}{{\left(\frac{{TF}}{{K}_{{TF}}}\right)}^{N}+1}+{A}_{{Target}}\right)\frac{1}{\left(1+{\left(\frac{{HIF}}{{K}_{H}}\right)}^{N}\right)}-{\alpha }_{{Target}}\cdot {Target}$$

Where:

*HIF*: Concentration of HIF-1.

*TF*: Concentration of the transcription factor.

*Target*: Concentration of the target gene product.

${K}_{H}$: Dissociation constant, indicates the concentration of HIF-1 at which the transcriptional response is at half-maximum.

${K}_{{TF}}$: Dissociation constant for TF at the target gene.

${\alpha }_{{TF}}$: Degradation rate of TF.

${\beta }_{{TF}}$: Maximum production rate of TF.

${\alpha }_{{Target}}$: Degradation rate of the target gene.

${\beta }_{{Target}}$: Maximum production rate of the target gene influenced by TF.

${A}_{{Target}}$: Additional activation of the target gene independent of TF.

N: Hill coefficient, describes the cooperativity of HIF-1 binding.

It can be observed that the silencing effect in Eq. (2) is multiplicative to the HIF-1 driven enhancer. Physiologically, it pertains to the effect of HIF-1 and the induced TF to be transcriptionally synergistic, or cooperative^30^. Exploring IFFLs with the target gene with an independent inhibitory term, we obtain Eq. (3).

3 $$\frac{{dTarget}}{{dt}}={\beta }_{{Target}}\cdot \left(\frac{{\left(\frac{{TF}}{{K}_{{TF}}}\right)}^{N}}{{\left(\frac{{TF}}{{K}_{{TF}}}\right)}^{N}+1}+\frac{{A}_{{Target}}}{1+{\left(\frac{{HIF}}{{K}_{H}}\right)}^{N}}\right)-{\alpha }_{{Target}}\cdot {Target}$$

The additive silencing by the HIF-1 driven silencer is also modeled here, which gives rise to a very different phase diagram, wherein even larger parameter regimes show cycling hypoxia-driven target gene expression.

### IFFL dynamics can generate gene expression responding specifically to cycling or intermediate hypoxia

**Fig. 4:**
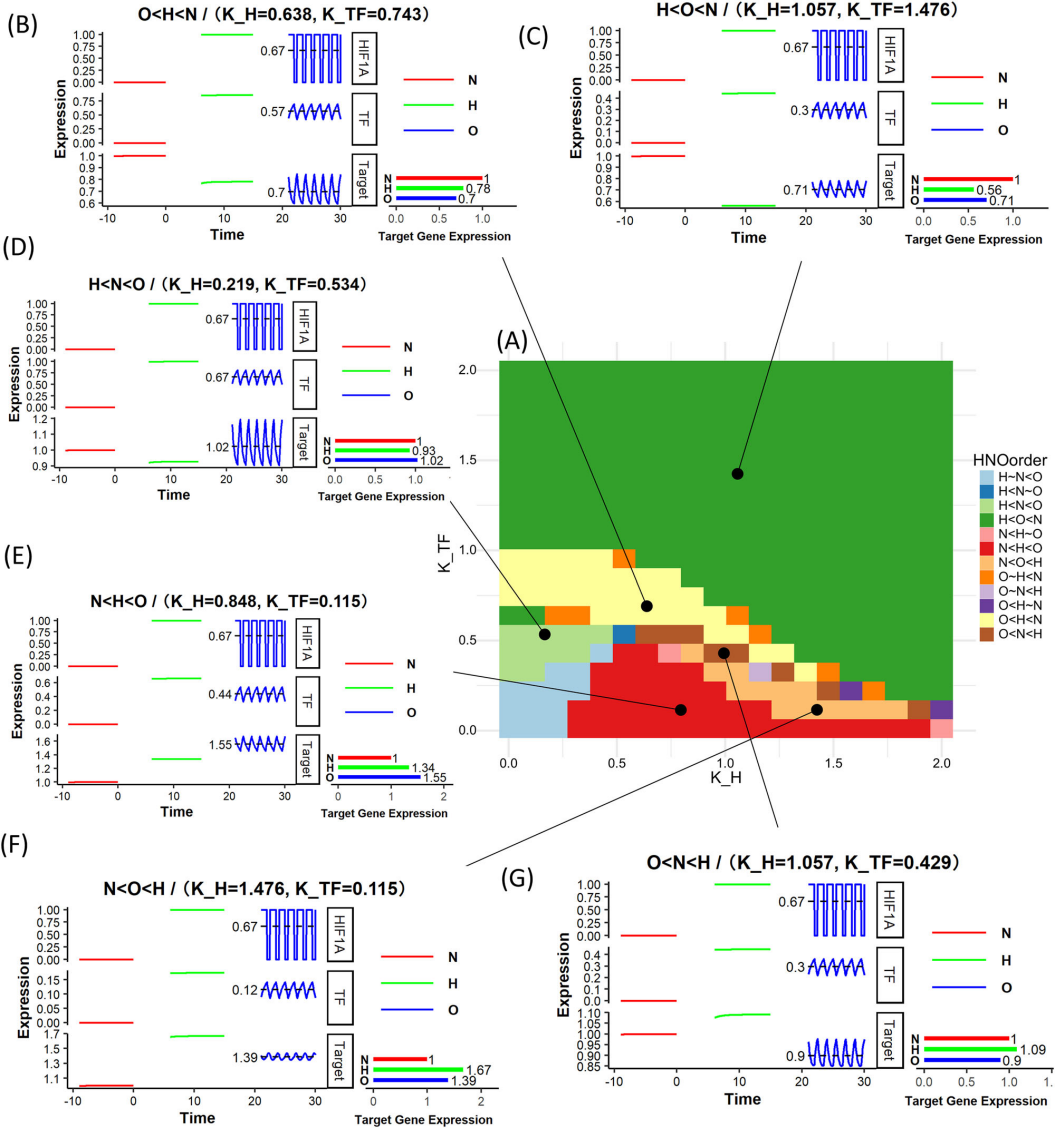
IFFLs with additive effect of HIF-1 and HIF-1-activated TF on target gene expression show cycling/oscillatory hypoxia-specific patterns. Phase diagram and example dynamics of IFFL Type 3 circuit with additive inhibition (Eqs. 1 and 3). **A** Phase diagram showing the relative order of the target gene expression in Normoxia (N), Hypoxia (H), and Oscillatory Hypoxia (O). **B**–**G** Example dynamics of the driving HIF1A that is set at 1 for hypoxia, 0 for normoxia, and pulsating 1 h of hypoxia and half an hour of normoxia for oscillatory hypoxia, the intermediate transcription factor, and the Target gene. All gene expression values are in arbitrary units. H, N, O order pattern given only in terms of the target gene expression.

We sought to explore if a) our IFFL model can generate target gene expression that responds specifically to cycling hypoxia, and b) the parameter ranges that would be required for such emergent responses. We numerically solved the differential equations based models of IFFLs described above in R to simulate gene expression patterns in different hypoxic conditions and parameter ranges. We simulated the IFFL models both under multiplicative (Fig. 3) and additive (Fig. 4) inhibition, shown here for one of the IFFL sub-types (*type III*). We independently varied the HIF-1 dissociation constant (${K}_{H}$) and the intermediary transcription factor dissociation constant (${K}_{{TF}}$). For each parameter set, we classify the oscillation-specific responses according to the order of target gene expression under stable hypoxia (H), normoxia (N), and cycling/oscillatory hypoxia (O) (Figs. 3A, 4A).

HIF-1 is assumed to follow a cyclical expression pattern, consisting of 1 h of sustained high expression followed by 0.5 h of degradation, to mimic oscillatory hypoxia conditions, as shown in Figs. 3B–F and 4B–F. To explore the relationship between target gene expression and the sensitivity of the system to hypoxia (${K}_{H}$) and TF activity (${K}_{{TF}}$), we simulated each of the four IFFL types, separately for multiplicative, and additive silencing by HIF-1 (Supplementary Information 1 (*Modeling continuous HIF-1a dynamics under oscillatory oxygen levels*)). For the simulations, we set the initial condition for both the transcription factor (TF) and the target gene to zero. All parameter values for the simulations are provided in Supplementary Information 1 (*Model circuits and parameters*). Phase diagrams identify distinct oscillatory/cycling hypoxia specific gene expression patterns. In the regimes where the inhibitory arm dominates, we observe, as in Fig. 3B and Fig. 4C, that the target gene expression is the lowest under hypoxia, intermediate in oscillatory hypoxia, and greatest under normoxia (H < O < N pattern). Similarly; when the activating arm dominates, as in Figs. 3E and 4F, we obtain the opposite, but expected pattern of N < O < H. However, for some parameter combinations wherein the effect of the activating and inhibiting arms have comparable strengths but respond with different sensitivities or timing due to the nonlinearities of Eqs. 1–3, we observed the surprising behavior that the target gene expression under cycling hypoxia is either greater than under both hypoxia and normoxia (H < N < O) (Figs. 3D, 4D), or (N < H < O) (Figs. 3E, 4E), or lesser than both, as in Figs. 3C, 4B (O < H < N), Figs. 3F, 4G (O < N < H). For this IFFL circuit, within the same parameter range, the additive inhibitory model shows a larger regime of cycling hypoxia-specific expression (Fig. 3) than the multiplicative model (Fig. 4).

**Fig. 5:**
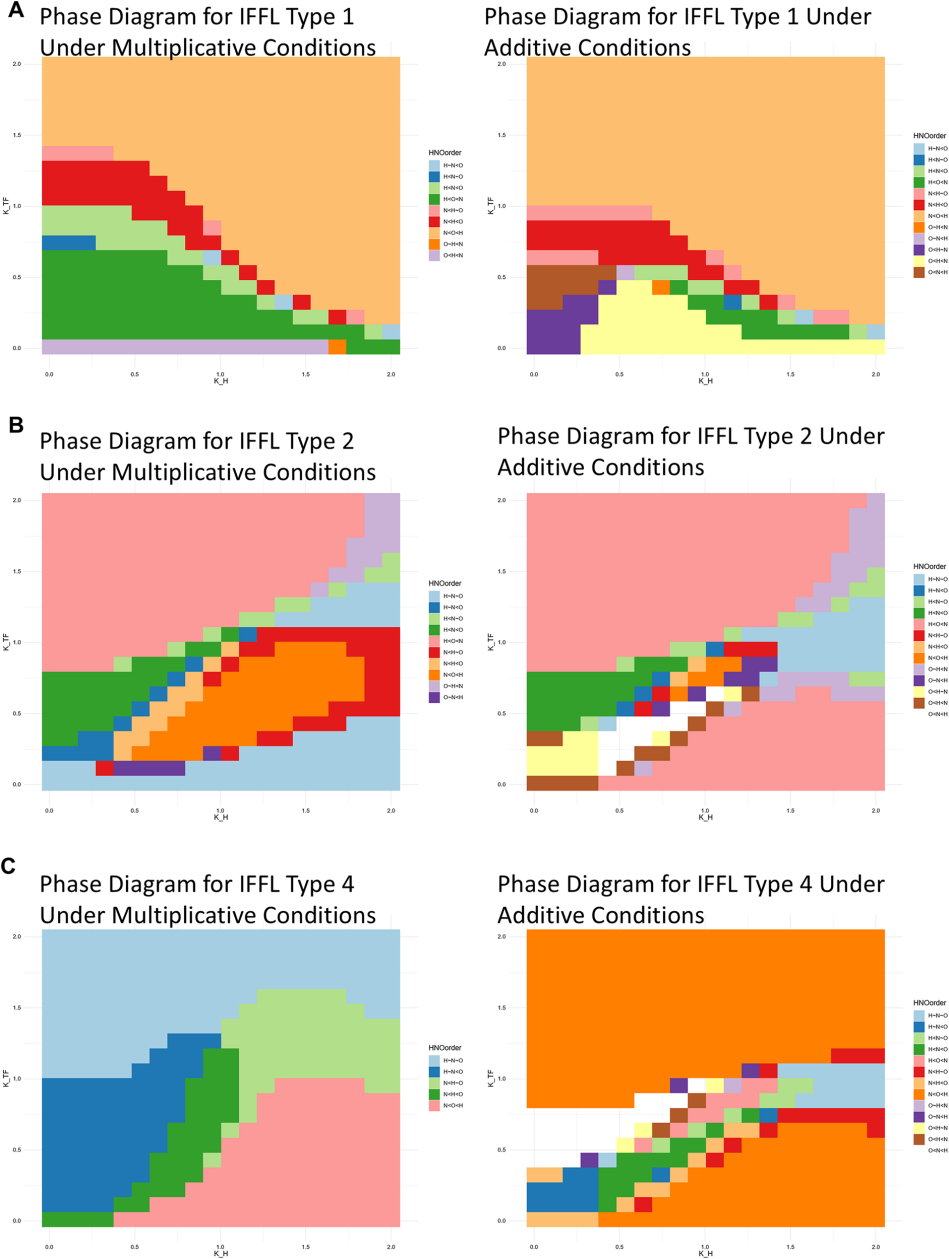
Both multiplicative and additive IFFLs show large parameter regimes with plausible oscillation-specific gene expression patterns. Phase diagrams showing the H, N, O order for target gene expression under hypoxia, normoxia, and cycling/oscillatory hypoxia conditions for IFFL circuits of Type 1 (**A**), Type 2 (**B**), and Type 4 (**C**). The first column simulates under multiplicative inhibition (Eqs. 1 and 2), while the second column simulates the circuits under additive inhibition (Eqs. 1 and 3).

We also computed similar phase diagrams for other IFFL circuits (Fig. 5), showing substantial regimes of cycling hypoxia specific behavior (O < N < H, O < H < N, N < H < O, or H < N < O). Overall, our ODE based IFFL models demonstrate that cycling hypoxia-specific gene expression patterns can emerge. Detailed equations for the other simulated IFFL circuits are provided in Supplementary Information 1*(Model circuits and parameters)*. Furthermore, in IFFL models where HIF-1 and a HIF-1–induced transcription factors regulate the target gene through additive mechanisms, the parameter regimes for oscillation/cycling hypoxia specific gene expression was much larger than the multiplicative models. In addition, we show in Supplementary Information 1*(Steady state analysis of IFFL circuits)* that the IFFL circuits shown here can also produce these cycling or intermediate hypoxic specific responses by responding to the time-averaged hypoxic signal lying in between stable hypoxia and normoxia.

While the simulations performed here assumed discrete HIF1 dynamics in order to arrive at the minimal IFFL model, we also showed in Supplementary Information 1 (*Modeling continuous HIF-1a dynamics under oscillatory oxygen levels*) that assuming continuous HIF1 dynamics driven by an external oxygen input similarly shows parametric regions cycling/oscillation specific gene expression.

### HIF-1 recruits p53 and Notch1 in IFFL gene regulatory units to drive cycling hypoxia-specific gene expression

We experimentally tested if IFFLs formed by HIF-1 are responsible for oscillatory hypoxia–specific gene expression. Being a master transcriptional regulator^31^, HIF-1 transcriptionally activates a large number of genes, many of which themselves encode transcription factors. Based on a large number of previous reports, p53, and Notch1 were considered to be putative HIF-1 activated TFs, which could directly or indirectly influence gene expression of HIF-1 transcribed genes. Both p53, encoded by the TP53 gene, and Notch1 are important players in various cancers, and their involvement with HIF- is well established^32–34^. HIF-1 can reciprocally regulate p53, and also activates Notch1 by interacting with the intracellular domain of Notch (NICD)^33,35^.

To test if p53 and Notch1 can participate in IFFLs driven by HIF-1 and therefore regulate oscillatory/cycling hypoxia-specific gene expression, we silenced TP53 and NOTCH1 genes in MDA-MB-231 cells, and sequenced RNA after the protocol adopted in normoxia (N), hypoxia (H), and cycling hypoxia (O). Filtering for genes that showed significant changes among the N, H, and O conditions in wild-type cells, but whose patterns were disrupted in TP53- or NOTCH1-silenced conditions, we found several genes whose cycling hypoxia-specific expression patterns were dependent on the participating TF. In case of TP53, we found all 4 classes of cycling/oscillatory hypoxia-specific genes to be TP53 dependent in their unexpected expression patterns.

**Fig. 6:**
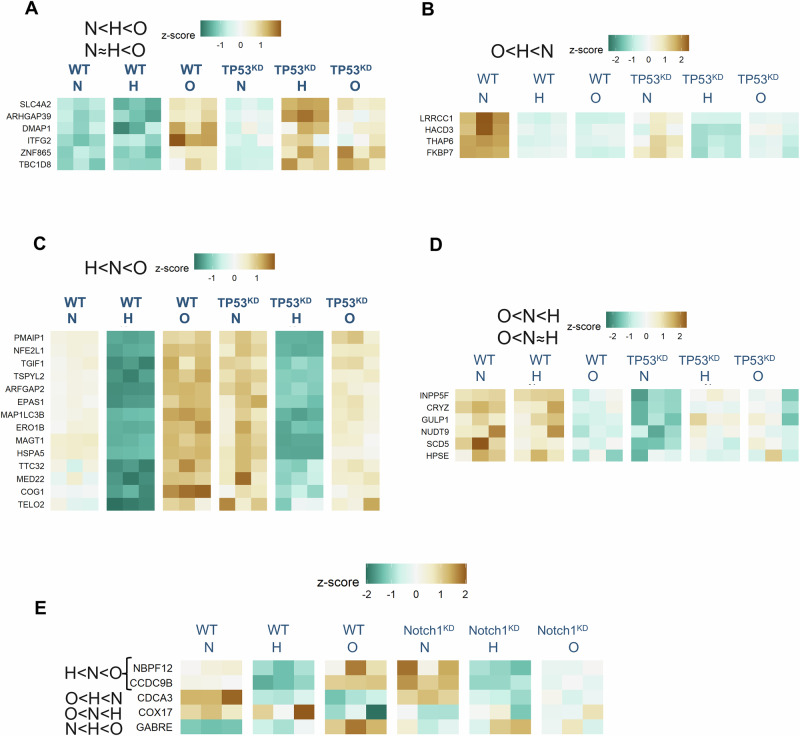
Target genes showing both oscillatory specific gene expression, and whose gene expression pattern can be explained by a gene circuit mediated by TP53 or Notch1. **A** N < H < O oscillatory specific pattern genes, whose oscillatory specific pattern is lost on TP53 knock down. **B** O < H < N oscillatory specific pattern genes, whose oscillatory specific pattern is lost on TP53 knock down. **C** H < N < O oscillatory specific pattern genes, whose oscillatory specific pattern is lost on TP53 knock down. **D** O < N < H oscillatory specific pattern genes, whose oscillatory specific pattern is lost on TP53 knock down. **E** Oscillatory specific pattern genes, whose oscillatory specific pattern is lost on Notch1 knock down.

Several genes which showed patterns where cycling hypoxia had a directionally congruent, but a more extreme effect than stable hypoxia were found to be TP53 dependent (Fig. 6A, B). Several of these TP53 dependent genes have potential role in cancer malignancies. These include SLC4A2, which encodes AE2, a bicarbonate-sodium exchanger which is upregulated in breast cancer contributing the metastasis^36^. We have previously also reported that oscillatory hypoxia results in increased expression of several ion channels and symporters^10,37^. Similarly, another key TP53 IFFL gene was ARHGAP39, a RHOA GTPase^38^ and ITFG2 which encodes Integrin alpha-2 implicated in breast cancer metastasis and poor survival outcomes^39^. Similarly, TBC1D2 which encodes a gene in the TBC domain family promotes E-cadherin degradation, and has a poor prognosis for ovarian cancer^40^. O < H < N pattern genes which were found to be TP53 dependent were mostly related to lipid metabolism, including FKBP7 a fatty acid binding protein, and HACD3 encoding for 3-hydroxyacyl-CoA dehydratase 3, as well as other pro-invasive genes THAP6 and LRRCC1, both associated with increased metastasis in various cancers (Fig. 6C).

Interestingly, the genes which showed an opposite effect on expression in cycling hypoxia vs stable hypoxia were found to be more frequently TP53 dependent (Fig. 6D, E). Among the genes which had H < N < O patterns in wild-type MDA-MB-231 and were found to be p53 dependent, most have been reported extensively for their role in TNBC (Fig. 6D). Interestingly, many of these genes were associated with ER and Golgi based protein folding, or lipid metabolism, or those associated with autophagy, dysregulation of all critical in cancer progression. These included NFE2L1 which encodes the TF Nrf1, a key regulator of glucose and lipid metabolism^41,42^, MAP1LC3B encoding LC3B a key regulator of autophagy^42,43^, ERO1B encoding endoplasmic reticulum oxidoreductase 1 beta and strongly linked to TNBC malignancy and poor survival^44^, HSPA5 encoding heat shock protein family A member 5, and COG1 encoding a protein part of the conserved oligomeric golgi complex. In addition, EPAS1, which encodes HIF-2a and is linked to breast cancer prognosis was also found to be a gene which expresses in cycling hypoxia in a TP53-dependent manner^45^. Similarly, genes which increased in hypoxia, but decreased in stable hypoxia in wild-type cells but not in TP53 silenced cells included HPSE encoding heparinase, which is elevated in serum of breast cancer patients and has poor prognosis^46^, as well as NUDT9 which modulates HIF-1 activity (Fig. 6E)^47^.

We also tested the hypothesis of HIF-1 driven IFFLs with another TF, Notch1, which is activated by HIF-1 itself and acts as a transcriptional regulator of many genes (Fig. 6F). Using a similar methodology with TP53 KD cells in N, H, O, conditions, we identified a few genes that Notch1 may be regulating as part of IFFL with HIF-1. Again, we found genes that were upregulated under cycling hypoxia but downregulated under stable hypoxia, with this pattern being dependent on Notch1. These genes, including *NBPF12*, *CCDC9B*, and *GABRE* (which encodes a subunit of the GABA receptor), are known to play roles in breast cancer progression. Overall, our experimental results not only identify, but demonstrate a causal role for two key HIF-1-regulated transcription factors in driving gene expression patterns that are specific to oscillatory/cycling hypoxia, at times even showing responses opposite to those under stable hypoxia.

While our perturbation experiments support a functional IFFL relationship involving HIF-1, p53, or Notch1, we have not experimentally confirmed direct TF binding at target gene promoters. Further studies using ChIP-seq, promoter reporter assays, or other experiments could validate the physical regulatory interactions.

## Discussion

The role of hypoxia is well established in nearly all aspects of tumor biology. Development of hypoxic cores in the growing tumors, wherein the vascular perfusion is unable to match the need for molecular oxygen, results in stabilization of HIF-1, a heterodimeric protein complex^2,6^. HIF-1 is a master transcriptional regulator, and its stabilization results in a remarkable change in cellular state, influencing many aspects of cellular metabolism, proliferation, differentiation, migration, and interaction with other cells. However, HIF-1 has also been shown to inhibit cell cycle progression, yet cancers continue to grow in hypoxia. We partly explained this conundrum by showing that fluctuations in HIF-1 stability, and therefore in downstream transcription, may provide the opportunity for cells to continue to divide even in hypoxia^14^. HIF-1 can exhibit oscillations in its transcriptional activity due to activation of chaperone-mediated autophagy due to excess lactate in the hypoxic tumor milieu. In addition, various reports have shown that hypoxia itself is not stable in the tumor, and may cycle, particularly in the intermediate regions between the vascular and avascular regions^8,9^.

Here, we elucidate the plausible mechanism of specific gene expression pattern driven by endogenous fluctuations in HIF-1 activity, or hitherto less understood, or even appreciated aspect of tumor biology, cycling or oscillatory hypoxia^7^. Our RNAseq data on various cell types have revealed many genes which exhibit a cycling hypoxia specific gene expression pattern^10,14^. Ordinarily, cycling hypoxia signal would be averaged over many time periods to be between the normoxic, and hypoxic range, and indeed, most genes exhibit this pattern. However, we found that for many genes, cycling/oscillatory hypoxia can increase the effect of hypoxia itself, even though the averaged O_2_ levels are lower when integrated over many cycles. Surprisingly, we also found that cycling/oscillatory hypoxia had an opposite effect on many downstream genes compared to stable hypoxia. These oscillation-specific patterns are not possible to be explained by conventional linear regulatory mechanisms, suggesting more complex regulatory models.

In this work, we chose the Incoherent Feed Forward Loop (IFFL) to model oscillatory input signals, and their effect on the target gene expression as plausible models for multiple reasons^20^. IFFLs are simple modules, consisting of just 3 factors, necessitating the inclusion of only one new species in addition to HIF-1 and the target gene. IFFLs are also a gene regulatory motif, indicating that they are evolutionarily selected owing to many selective advantages. As HIF-1 is a master transcriptional regulator, which regulates expression of many genes that themselves encode TFs, there are opportunities for IFFLs formation due to many HIF-1 targets. Finally, in certain parameter regimes, IFFLs can allow discrimination of variable and stable input signals^27^, and the nonlinearities of each transcriptional interaction in the IFFL can also give rise to gene expression tuned to intermediate hypoxic signals Supplementary Information 1*(Steady state analysis of IFFL circuits)*. It is important, to note, however, that there are other mechanisms by which the transcriptional responses of some oscillation genes may be produced. Our motivation is that the exploration of the IFFL mechanisms here will lead to the revelation of specific intermediary components that could be further explored. Indeed, we tested the effect of Notch1 and p53 on gene expression patterns in N H and O conditions. We therefore mathematically modeled IFFLs to test the possibility of cycling/oscillation HIF-1 driven gene expression. All 4 IFFL models, integrating either the additive or synergistic/multiplicative combination of the direct effects of HIF-1 and HIF-1 induced TFs on the target gene, revealed large parameter regimes where oscillation-specific gene expression was plausible. Interestingly, we found all specific subtypes of oscillation/cycling hypoxia-driven gene expression patterns that we had observed in experimental data.

While our study provides insights into the potential regulatory roles of IFFLs under varying hypoxic conditions, it has several limitations. First, our modeling framework uses deterministic ordinary differential equations, which do not capture stochastic fluctuations or spatial heterogeneity of biological systems. Second, we focus on a single cycling hypoxia protocol (1 h hypoxia, 0.5 h reoxygenation), which may not fully represent the diversity of oxygen dynamics in tumor microenvironments. Third, our experimental validation is based on in vitro cell line data under controlled oxygen conditions, which may differ from in vivo contexts. Although our experiments were performed under standard atmospheric oxygen (~21%), which exceeds physiological tumor oxygen levels (~5%), our focus is on the *relative dynamics* between stable and fluctuating hypoxia, and a reductive in-vitro system that amplifies the hypoxic response signal. Future studies could explore a broader range of oxygen fluctuation patterns, incorporate cell-cycle coupling, and validate findings using single-cell or spatial transcriptomic data from clinical samples.

Finally, we identified two key TFs which could potentially participate in HIF-1-driven IFFLs to discriminate between variable and stable HIF-1 signals, with a profound effect on downstream gene expression. Both p53, and Notch1 are TFs critical in cancer progression, including breast cancer, and are known to be activated by HIF-1. Using gene perturbation of either TF encoding genes and subjected cells in normoxia, hypoxia, and cycling hypoxia conditions, we discovered several genes which are cycling/hypoxia specific in TP53, or Notch1 dependent manner. Remarkably, a large number of these genes, which putatively participate in IFFLs with HIF-1 and p53 (or Notch1) are pro-oncogenic genes, associated with poor prognosis in breast cancers, as well as other cancers.

More broadly, oscillatory signals are common across multiple scales of biology. Circadian rhythms, both systemic and the transcriptional clock regulators in the nucleus, endocrinal signals like the pulsating Gonadotropin releasing hormone production which drives the hyopthalamic–pituitary gonadal axis, as well as calcium waves within the cytoplasm are many examples of rhythmic, or cycling signals, critical for various physiological functions. These cycling signals are processed in a specific way to elicit specific signaling, or gene expression responses. Their downstream processing itself could be complex, resulting in cyclical responses at a different frequency, e.g., hourly pulses of GhRH ultimately regulate the monthly ovulatory and menstrual cycles. Oscillations are certainly more common in signaling than are studied. Our approach could be used to model how oscillatory signals are processed downstream at the transcriptional level, presenting a methodological framework to identify the intermediaries which result in specific types of gene expression patterns. It is important to note that this circuit, which shows cycling-specific reponse, can also show a response tuned to the time averaged oscillatory signal. Thus such nonlinear circuits could be key to the response to many such dynamic or intermediate signals. As many gene express in response to oscillatory signals, identifying additional transcription factor partners may provide new opportunities to therapeutically target transcription associated with various pathologies, including cancer. Finally, our work also pave a path to molecularly target cycling hypoxia specific gene expression by providing the putative transcriptional machinery underlying their expression.

## Methods

### Computational modeling and simulation of IFFL circuits

To investigate the gene regulatory behavior of Incoherent Feed-Forward Loop (IFFL) circuits under varying oxygen conditions, we developed a mathematical model based on ordinary differential equations (ODEs) representing the interactions among HIF-1α (HIF1A), an intermediate transcription factor (TF), and a downstream target gene, as shown in Eqs. 1–3 and in the supplementary methods. Simulations were implemented using the deSolve package in R^48^.

For the generation of phase diagrams, we independently varied the dissociation constants for HIF1A and the intermediate TF (${K}_{H}$ and${K}_{{TF}}$) in a range from 0.01 to 2 (in normalized units). This range was selected to ensure both mathematical stability and biological relevance, avoiding division by zero and preserving the numerical robustness of the simulations. Each parameter was discretized into 20 evenly spaced intervals. Unless otherwise noted, baseline parameter values, including degradation rates and maximum production rates of TF and the target gene (${\alpha }_{{TF}}$, ${\alpha }_{{Target}}$, ${\beta }_{{TF}}$, ${\beta }_{{Target}}$), were set to 1.

To capture gene expression dynamics under normoxia (N), stable hypoxia (H), and cycling/oscillatory hypoxia (O), we selected representative points from the phase diagram for detailed time-series analysis. Simulations were conducted across a time interval from −15 to 30 arbitrary units with a resolution of 0.05. The intervals −15 to 0, 0 to 15, and 15 to 30 corresponded to normoxia, stable hypoxia, and cycling hypoxia conditions, respectively. To highlight the steady-state expression dynamics in each condition, we computed and visualized mean gene expression levels during the final 9 units of each interval: (−9, 0) for normoxia, (6, 15) for hypoxia, and (21, 30) for cycling hypoxia.

During the oscillatory phase, we modeled environmental cycling with alternating hypoxic (low) and normoxic (high) conditions. Each cycle consisted of 1 h of hypoxia followed by 0.5 h of normoxia, yielding a total cycle length of 1.5 h, which approximates the experimental temporal dynamics applied in vitro. This asymmetric design is consistent with physiological observations that reoxygenation in tumor tissue tends to be slower than deoxygenation due to limited vascular recovery and diffusion capacity.

### Transcriptional profiling of cancer cells

MDA-MB-231, a breast cancer cell line, was subjected to normoxia, stable hypoxia, and cycling hypoxia (1 h of hypoxia and 0.5 h of normoxia). We also obtained conducted CRISPR-mediated silencing of TP53 and NOTCH1 in MDA-MB-231 cells to obtain three different cell conditions: wild type (WT), TP53KD, and NOTCH1KD. This resulted in 3 cell conditions, with 3 oxygen treatments, and 3 technical replicates, with a total of 27 samples that were sequenced for mRNA profiling on the Illumina platform after standard mRNA library preparation. The obtained paired-end mRNA reads were aligned to the human genome using HISAT2^49^, and concordantly aligned counted against the standard gene models using featureCounts^50^. The sample x gene matrix was analyzed for differential expression using DESeq2^51^ on the R platform. Significance was defined as an adjusted p-value < 0.05. Genes exhibiting differential expression^49^ across normoxic, hypoxic, and oscillatory conditions were identified and categorized by their N-H-O expression relationships.

To infer the regulatory role of TP53 or NOTCH1, we examined expression shifts in their respective knockout conditions. If a target gene Z showed a statistically significant increase in TP53KD or Notch1KD relative to WT, the repressive role of the corresponding transcription factor was inferred. Conversely, a decrease in expression indicated an activating relationship. Using this approach, we constructed putative HIF1A-TP53-target and HIF1A-Notch1-target regulatory loops based on congruent directional changes under HIF1A activation (assessed via expression in WT hypoxia versus normoxia).

## Supplementary information

Supplementary information

Supplementary Data 1

## Data Availability

The RNAseq data are available in NCBI Gene Expression Omnibus (GEO) database: Accession record: GSE292924. Code is deposited in GitHub: DOI: 10.5281/zenodo.15069799.
