# Supplementary material for: Hif-1 responsive IFFLs to explain specific transcriptional responses to cycling hypoxia in cancers
Source: NPJ Syst Biol Appl. 2025 Nov 24;11:136. doi: 10.1038/s41540-025-00612-z (PMC12660717; doi:10.1038/s41540-025-00612-z)
Supplement: Supplementary file 1 — Supplementary information [file 41540_2025_612_MOESM1_ESM.pdf]

# Supplementary Information

## Supplementary Note 1. Modeling Continuous HIF-1$\alpha$ Dynamics under Oscillatory Oxygen Levels

### 1.1. HIF-1$\alpha$ Dynamics Model based on Oxgen level

As HIF-1$\alpha$ dynamics can be modeled as a function of oxygen levels using a Hill-type inhibition function, we generated an ordinary differential equation of HIF-1$\alpha$ with respect to time t.

$$\frac{dH}{dt} = \frac{H_{max}}{1+\left(\frac{O_2}{K_{O_2}}\right)^m} - \alpha_H \cdot H \qquad (1)$$

Where:

$H$: Concentration of HIF-1$\alpha$.
$H_{max}$:Maximum rate of HIF-1$\alpha$ production under hypoxic conditions
$O_2$: Oxygen concentration.
$K_{O_2}$: Half-saturation constant for oxygen, i.e., the oxygen level at which HIF-1$\alpha$ production is at 50% of its maximum.
m: Hill coefficient, representing the cooperativity of oxygen's inhibitory effect.
$\alpha_H$: Degradation rate constant of HIF-1$\alpha$.

#### 1.1.1 Parameter Estimation

We modeled the temporal dynamics of HIF-1$\alpha$ protein under hypoxic and normoxic conditions using the Eq. (1). To uniquely determine the four parameters $H_{max}$, m, $K_{O_2}$ , $\alpha_H$, we adopted the following approach:

$H_{max}$was set to 1 in normalized units, consistent with prior modeling approaches. The Hill coefficient m was set to 4, a common choice reflecting a steep oxygen sensitivity. In our model, the extracellular oxygen concentration $O_2(t)$ was normalized to a range between 0 and 1, where 1 corresponds to atmospheric oxygen tension (21% $O_2$), and 0 represents

complete anoxia. Under this normalization, a hypoxic oxygen level of 1% corresponds to: $O_{hyp}$=1/21≈0.048≈0.05, and a normoxic level of 21% corresponds to:$O_{norm}$=21/21=1.

To estimate the remaining two parameters $K_{O_2}$ and $\alpha_H$, we incorporated two time-course constraints derived from experimental trends reported in the literature:

a. **Accumulation under hypoxia:** Experimental studies have shown that HIF-1$\alpha$ protein levels gradually accumulate under sustained hypoxia and typically reach near-maximal levels within 2 to 4 hours[1,2]. Based on this range, we assumed that HIF-1$\alpha$ increases from 0 to 0.99 over a 3-hour period when $O_2$ = 0.05.

b. **Degradation under normoxia:** Following reoxygenation, HIF-1$\alpha$ protein is rapidly hydroxylated and degraded via the proteasomal pathway. Previous observations indicate that HIF-1$\alpha$ decays to near-baseline levels within 15–60 minutes under normoxic conditions[3,4]. We therefore assumed a decay from 1.0 to 0.01 over 1 hour when $O_2$ = 1.

Thus, the parameters $K_{O_2}$ and $\alpha_H$ were estimated by solving a nonlinear system that enforces the model to satisfy two time-course constraints:

a. Under constant hypoxia ($O_2$=0.05), HIF-1$\alpha$ rises from 0 to 0.99 within 3 hours (i.e., H(0) = 0, H(3) = 0.99).
b. Under constant normoxia ($O_2$=1), HIF-1$\alpha$ decays from 1.0 to 0.01 within 1 hour (i.e., H(0) = 1, H(1) = 0.01).

These two conditions were incorporated into a root-finding system, where the unknowns $K_{O_2}$ and $\alpha_H$ were estimated by numerically solving the ODE forward and minimizing the deviation from the target values. The final estimated values were: $K_{O_2}$=0.213977≈0.21, $\alpha_H$=1.150356≈1.15.

Notably, the estimated value of $K_{O_2}$ corresponds to ~4.4% $O_2$, which lies within the known oxygen sensitivity range of prolyl hydroxylase domain proteins (PHDs) (3–5% $O_2$ or ~20–40 mmHg)[5]. PHDs are oxygen-dependent enzymes that hydroxylate proline residues on HIF-1$\alpha$, marking it for proteasomal degradation under normoxic conditions. Their enzymatic activity is highly sensitive to oxygen availability, making them key regulators of HIF-1$\alpha$ stability and justifying the inferred $K_{O_2}$ value in our model.

### 1.1.2. Time-Varying Oxygen Tension: Stepwise Input

Based on our experimental design, the oscillatory hypoxia condition consists of 1 hour of hypoxia followed by 0.5 hour of normoxia, repeated over a total duration of 48 hours. Therefore, the time-varying oxygen concentration $O_2(t)$ can be defined as:

$$O_2(t) = \begin{cases} O_{hyp} = 0.05\,, & if\ t\ mod\ 1.5\ <\ 1 \\ O_{norm} = 1\,, & if\ t\ mod\ 1.5\ \geq\ 1 \end{cases} \tag{2}$$

## 1.2. Simulation Results

Our simulation illustrates that although the $O_2$ input exhibits abrupt stepwise transitions, the response of HIF-1$\alpha$ is gradual due to delayed kinetics governed by the underlying differential equation. After approximately 3.5 hours, HIF-1$\alpha$ levels begin to oscillate within a confined range at each $O_2$ level (when $O_2$ = 0.05, H ≈ 0.406–0.719; when $O_2$ = 1, H ≈ 0.411–0.721), effectively compressing HIF-1$\alpha$ dynamics. This behavior reflects the emergence of a limit cycle induced by periodic oxygen input **(Supplementary Figure 1.A–B)**.

Importantly, this oscillation-specific behavior does not emerge from static input conditions but is instead driven by the dynamic nature of HIF-1$\alpha$ itself in response to periodic forcing. The inclusion of HIF-1$\alpha$ kinetics in the model is sufficient to generate this unique response, even under simplified stepwise oxygen changes. This highlights that dynamic regulation—rather than just steady-state input levels—can shape system behavior in a manner distinct from static normoxia or hypoxia.

### 1.2.1 Stepwise Oscillation in $O_2$ Leads to Gradual HIF-1$\alpha$ Adaptation with Limit Cycle Behavior

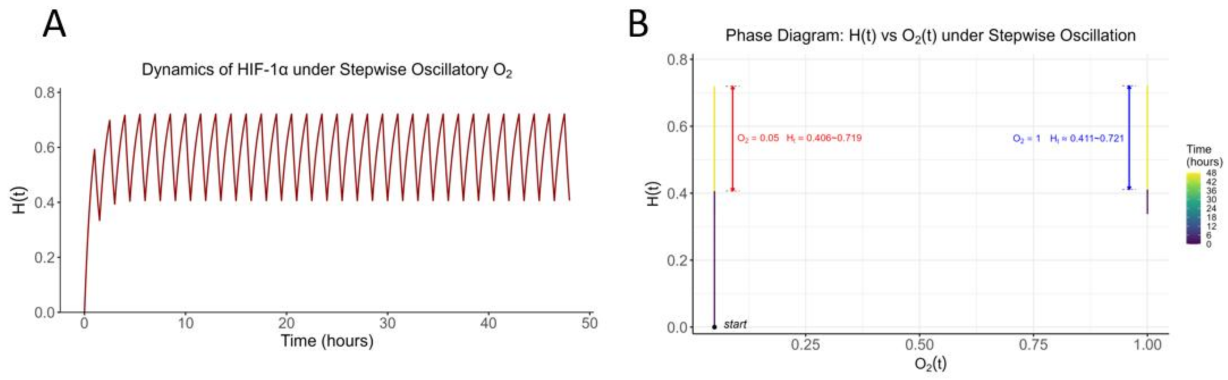


**Supplementary Figure 1.** Simulated dynamics of HIF-1$\alpha$ in response to stepwise oscillatory oxygen levels. (A) Time-course simulation of HIF-1$\alpha$ (H(t)) under repeated switching between hypoxia ($O_2 = 0.05$) and normoxia ($O_2 = 1$) every 1.5 hours. After an initial transient phase (~3.5 hours, not directly shown), HIF-1$\alpha$ levels settle into a stable oscillatory pattern. (B) Phase diagram showing H(t) as a function of $O_2$(t), colored by time. Vertical bars indicate the steady-state ranges of HIF-1$\alpha$ during each oxygen phase over the final 1.5-hour cycle.

### 1.2.2 Oscillatory Oxygen Induces Expression Patterns Beyond What Fixed Intermediate Oxygen Can Achieve

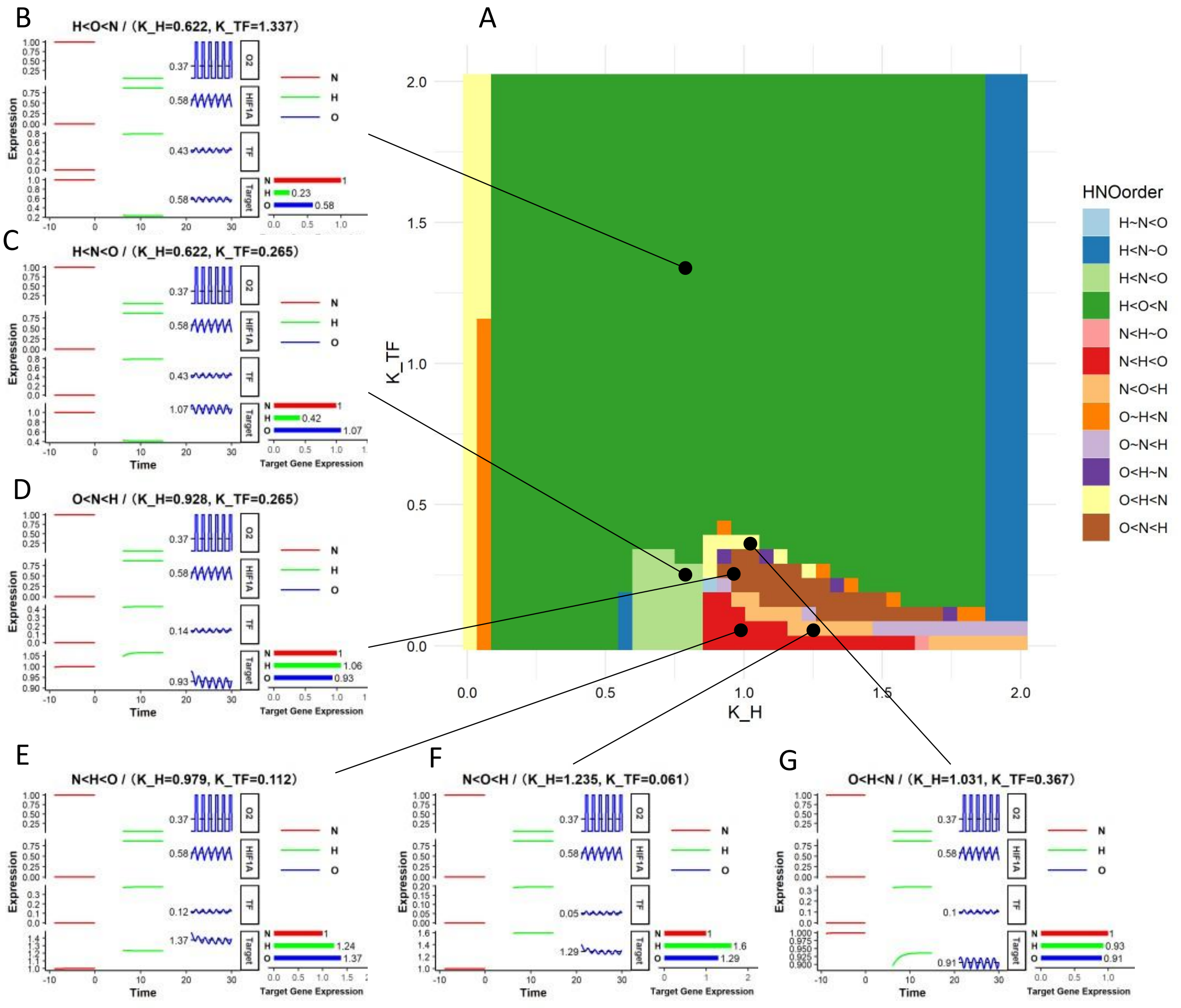


**Supplementary Figure 2. IFFLs responding to oscillatory oxygen inputs exhibit unique expression patterns not recapitulated by static normoxia or hypoxia.** Phase diagram and example dynamics of the IFFL Type 1 circuit driven by **stepwise oscillatory $O_2$ input** (defined as 1 hour of hypoxia at 0.05 followed by 0.5 hour of normoxia at 1.0, repeated every 1.5 hours).(A) Phase diagram showing the ordering pattern based on **target gene expression** in Normoxia (N), Hypoxia (H), and Oscillatory Hypoxia (O).(B–G) Example dynamic trajectories of the oxygen input, simulated HIF-1$\alpha$ expression, intermediate transcription factor (TF), and target gene expression under normoxia (N = 1.0), hypoxia (H = 0.05), and oscillatory hypoxia (O = stepwise cycling).

H, N, and O denote the **mean expression** levels of the target gene under the respective conditions. Each inset summarizes the expression distribution and average values.

# Supplementary Note 2. Steady State Analysis of the IFFL Circuit

## 2.1. Analytical expressions for TF and Target at steady state

To better understand how HIF-1$\alpha$ concentration modulates target gene expression, and whether intermediate oxygen levels can elicit stronger responses than those observed under normoxia or oscillatory hypoxia, we derived an analytical expression for the steady-state concentration of the target gene in the IFFL Type 3 motif (**Supplementary Figure 7**), denoted as Target$_{ss}$.

As described in the main manuscript, the system is governed by the following differential equations:

$$\frac{d\mathrm{TF}}{dt} = \frac{\left(\frac{H}{K_H}\right)^N}{\left(\frac{H}{K_H}\right)^N + 1} \cdot \beta_{TF} - \alpha_{TF} \cdot TF \qquad (3)$$

$$\frac{dTarget}{dt} = \beta_{Target}\left(\frac{\left(\frac{TF}{K_{TF}}\right)^N}{\left(\frac{TF}{K_{TF}}\right)^N + 1} + A_{Target}\right)\frac{1}{\left(1+\left(\frac{H}{K_H}\right)^N\right)} - \alpha_{Target} \cdot Target \qquad (4)$$

By setting the time derivatives to zero, we solve Eq. (3) to obtain the steady-state concentration of the transcription factor, denoted as $TF_{ss}$:

$$TF_{ss} = \frac{\beta_{TF} \cdot \left(\frac{H}{K_H}\right)^N}{\alpha_{TF} \cdot \left(\left(\frac{H}{K_H}\right)^N + 1\right)} \qquad (5)$$

Substituting $TF_{ss}$into Eq. (4) and again setting the derivative to zero yields the steady-state expression for the target gene:

$$Target_{ss} = \left(\frac{\beta_{Target}}{\alpha_{Target}}\right)\left(\frac{\left(\left(\frac{\beta_{TF} \cdot \left(\frac{H}{K_H}\right)^N}{\alpha_{TF} \cdot \left(\left(\frac{H}{K_H}\right)^N + 1\right) K_{TF}}\right)\right)^N}{1 + \left(\frac{\left(\beta_{TF} \cdot \left(\frac{H}{K_H}\right)^N\right)}{\alpha_{TF} \cdot \left(\left(\frac{H}{K_H}\right)^N + 1\right) K_{TF}}\right)^N} + A_{Target}\right)\left(\frac{1}{1 + \left(\frac{H}{K_H}\right)^N}\right) \qquad (6)$$

By evaluating $\text{Target}_{ss}$ as a function of key biochemical parameters, we are able to quantitatively assess how the IFFL topology and HIF-1$\alpha$-mediated signal integration give rise to non-monotonic and context-dependent gene expression dynamics.

## 2.2. Differential Sensitivity of Target Gene Expression to $K_H$ and $K_{TF}$ Reveals Non-Monotonic Regulatory Behavior

Based on the characteristics of the analytical solution, the behavior of the solution is primarily influenced by the parameters $K_H$ and $K_{TF}$. Therefore, we set the Hill coefficient N to 4, and all other secondary parameters were fixed at 1.

### 2.2.1. IFFLs can show peaks for intermediate values of the input

This paper attempts to explore the IFFL as the source for the cycling hypoxic response. While we have shown that IFFLs can show a response that is oscillation specific, here we explore whether this could still arise from HIF1a averaging. In the IFFL circuit, the time averaged HIF1a signal, being intermediate between normoxia and hypoxia, could still produce a target response that is more extreme than under normoxia and hypoxia. To this end, we use the steady state analytical expressions derived above, to plot the steady state response and its derivative for the range of HIF1a input, while varying $K_H$ and $K_{TF}$ in **Supplementary Figure 2**.

Specifically in **Supplementary Figures 2C-F,** we see that the response (Target gene expression) is <u>non-monotonous and in fact can show a peak at intermediate values of HIF1a</u>.

### 2.2.2. Low $K_H$ Enhances Target Activation and Accelerates Gene Response to HIF-1$\alpha$

**Supplementary Figure 3A** shows that when $K_H$ is low, the partial derivative $\frac{\partial \text{Target}_{ss}}{\partial H}$becomes increasingly negative as $H$ decreases, indicating accelerated upregulation of the target gene under low $H$. In other regions, the derivative remains flat and moderately negative, suggesting a steady inhibitory effect of increasing H, largely independent of both $K_H$ and $H$.

**Supplementary Figure 3B** shows that when $K_H$ is small, $\text{Target}_{ss}$ increases as $H$ decreases. As $K_H$ increases, this sensitivity diminishes, and the expression surface becomes flatter. This

suggests that at low $K_H$, the target gene is more responsive to HIF-1$\alpha$, even at low levels, whereas high $K_H$ dampens this effect.

### 2.2.3. Non-Monotonic Gene Activation Emerges under Low $K_{TF}$ Thresholds

We next explored the effect of the transcription factor activation threshold $K_{TF}$, which determines the sensitivity of the downstream gene to the transcription factor. During this analysis, $K_H$ was set to 1.

**Supplementary Figure 3C and 3D** display $\frac{\partial \text{Target}_{ss}}{\partial H}$from different viewing angles. At low $K_{TF}$, a non-monotonic response to $H$ is observed, with sensitivity peaking at intermediate HIF-1$\alpha$ levels and declining thereafter. The two perspectives complement each other in highlighting the shape and location of the sensitivity peak across parameter space.

**Supplementary Figure 3E and 3F** show that when $K_{TF}$ is low, the target gene expression exhibits a non-monotonic response to HIF-1$\alpha$, with a peak at intermediate $H$. As $K_{TF}$ increases, this peak gradually flattens, indicating that reduced sensitivity to the transcription factor diminishes the system's responsiveness to *HIF-1*$\alpha$.

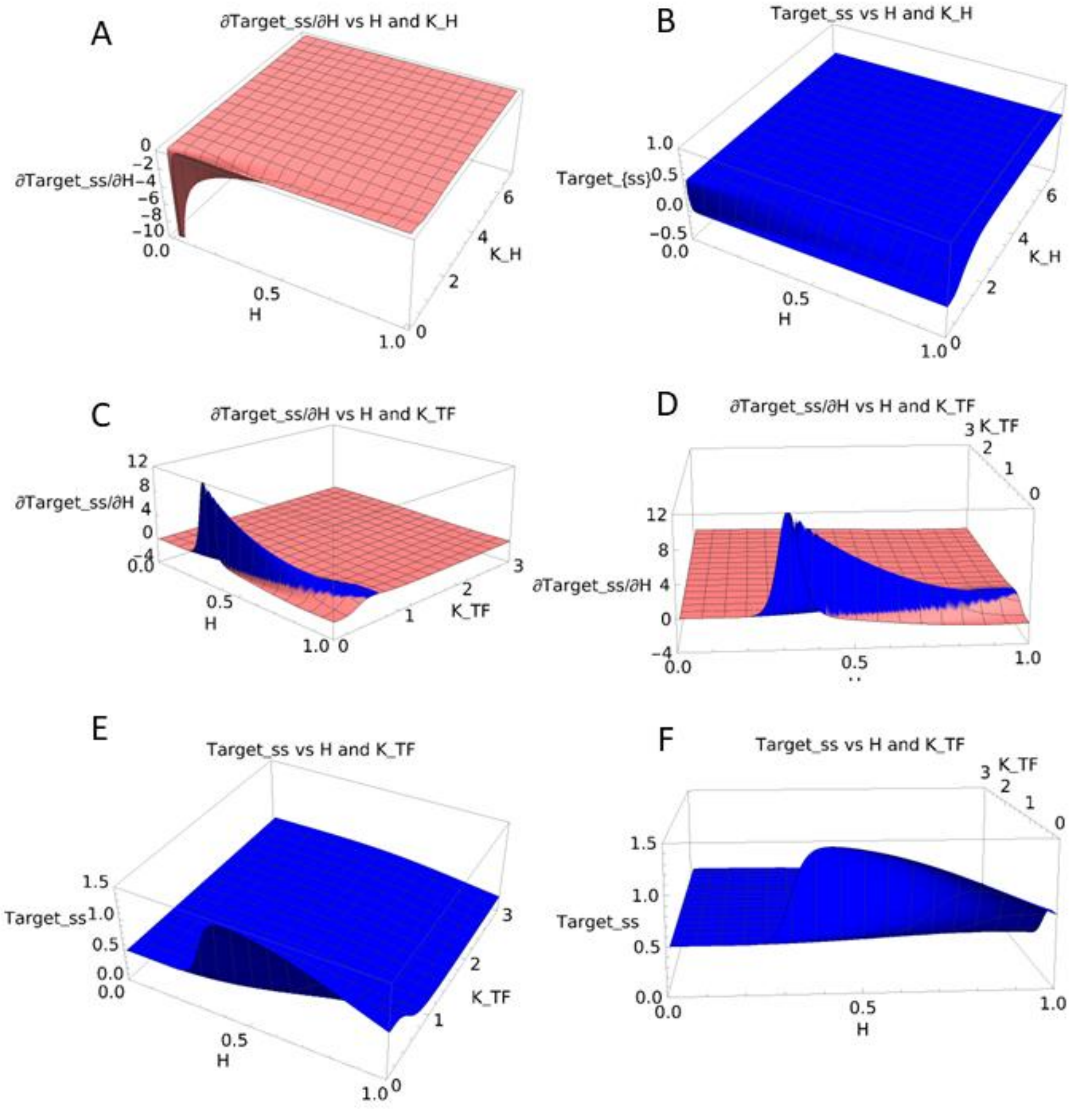


**Supplementary Figure 3. Non-Monotonic Target Gene Response to HIF1$\alpha$ Controlled by $\boldsymbol{K_H}$ and $\boldsymbol{K_{TF}}$.** (A) Partial derivative $\frac{\partial \text{Target}_{ss}}{\partial H}$plotted as a function of H and $K_H$, showing increasingly negative sensitivity at low $K_H$ and H. (B) Steady-state target expression Target$_{ss}$as a function of $H$ and $K_H$, showing increasing target expression as H decreases when $K_H$ is small; this effect diminishes as $K_H$ increases. (C-D) Partial derivative $\frac{\partial \text{Target}_{ss}}{\partial H}$ plotted against $H$ and $K_{TF}$ from two perspectives, showing a peaked sensitivity at intermediate H under low $K_{TF}$ .(E-F) Steady-state target expression Target$_{ss}$ as a function of H and $K_{TF}$ from two perspectives, also revealing a non-monotonic peak at intermediate H for low $K_{TF}$ , which flattens as $K_{TF}$ increases.

## 2.3. Global Parameter Scan

### 2.3.1 Scan Design Across Seven-Dimensional Parameter Space

To systematically evaluate the robustness of the observed non-monotonic behavior in our IFFL model, we conducted a comprehensive scan of 26,250,000 parameter combinations across biologically plausible ranges. The goal was to identify parameter regimes in which the steady-state expression of the target gene exhibits a local maximum at intermediate HIF-1$\alpha$ levels (H), relative to the two ends (low and high H values).

We varied the following seven parameters:

**Transcription factor parameters**

- $\alpha_{TF}$, $\beta_{TF}$ ∈ [0.1, 2.0], step 0.2
- $K_H$∈ [0.01, 7.0], step 0.1

**Target gene parameters**

- $\alpha_{Target}$, $\beta_{Target}$, $A_{Target}$ ∈ [0.1, 1.0], step 0.2
- $K_{TF}$ ∈ [0.01, 3.0], step 0.1

For each parameter set, we computed the steady-state expression of the target gene over a fine-grained range of HIF-1$\alpha$ and assessed whether the curve exhibited a local maximum or minimum between the two endpoints. We then categorized the curves according to their peak behaviors. The results showed that there are 1,507,800 (5.74%) combinations with intermediate maximum (i.e., max > both ends) and 2,041,425 (7.78%) combinations with intermediate minimum (i.e., min < both ends):

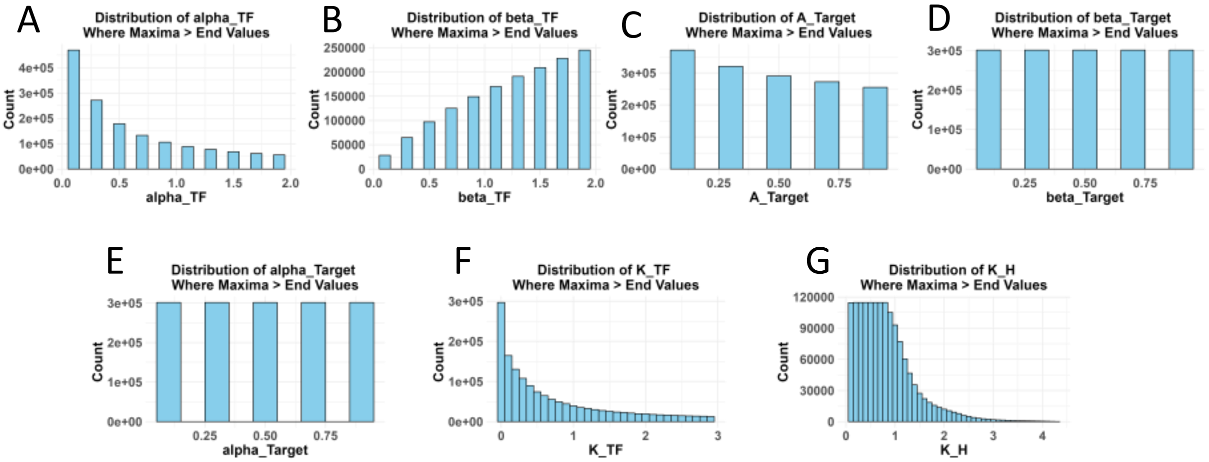


**Supplementary Figure 4. Distributions of model parameters in cases where the global maximum of the target expression exceeds both end values** (i.e., $\text{Target}_{max}$ > $\text{Target}_{start}$ and $\text{Target}_{end}$). Histograms are shown for all seven model parameters: (A) $\alpha_{TF}$ , (B) $\beta_{TF}$ , (C) $A_{Target}$ , (D) $\beta_{Target}$ , (E) $\alpha_{Target}$ , (F) $K_{TF}$ , and (G) $K_H$. Notably, intermediate-peak behavior is more likely to occur under specific parameter regimes, such as low $\alpha_{TF}$, $K_{TF}$ and $K_H$, and high $\beta_{TF}$, as evidenced by the skewed distributions in panels A, B, F and G.

These results indicate that non-monotonic profiles with intermediate peaks or troughs are indeed possible and occur in a notable fraction of the parameter space.

# <u>Supplementary Note 3.</u> Model Circuits and Parameters

## 3.1. Equation Summary for Four Types of IFFL Circuits

This section summarizes the ordinary differential equations used to define the four types of incoherent feedforward loop (IFFL) circuits considered in our model. Each circuit includes HIF-1$\alpha$ (denoted as H), a transcription factor (TF), and a downstream target gene. The expression of the target gene is jointly regulated by H and TF, with either multiplicative or additive integration logic. The Hill coefficient N governs the cooperativity of transcriptional regulation. While the main text focuses on Type 3 circuits, the full set of equations is provided here for completeness.

### 3.1.1. IFFL Type 1 Equations

In Type 1, HIF-1$\alpha$ activates both TF and the target gene, while the TF acts as a repressor of the target gene. The equations for multiplicative and additive integration are shown below.

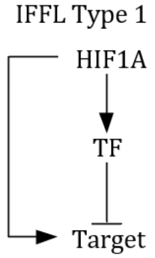


**Supplementary Figure 5. Schematic diagram of the Incoherent Feed-Forward Loop (IFFL) Type 1 motif.** In this motif, HIF1A activates both the intermediate transcription factor (TF) and the target gene, while the TF inhibits the target gene.

#### 3.1.1.1. Multiplicative Dynamics

$$\frac{d\mathrm{TF}}{dt} = \frac{\left(\frac{H}{K_H}\right)^N}{\left(\frac{H}{K_H}\right)^N + 1} \cdot \beta_{TF} - \alpha_{TF} \cdot TF \qquad (7)$$

$$\frac{d\mathrm{Target}}{dt} = \beta_{Target}\left(\frac{\left(\frac{H}{K_H}\right)^N}{\left(\frac{H}{K_H}\right)^N + 1} + A_{Target}\right)\frac{1}{1+\left(\frac{TF}{K_{TF}}\right)^N} - \alpha_{\mathrm{Target}} \cdot \mathrm{Target} \qquad (8)$$

### 3.1.1.2. Additive Dynamics

$$\frac{d\text{TF}}{dt} = \frac{\left(\frac{H}{K_H}\right)^N}{\left(\frac{H}{K_H}\right)^N + 1} \cdot \beta_{TF} - \alpha_{TF} \cdot TF \qquad (9)$$

$$\frac{d\text{Target}}{dt} = \beta_{\text{Target}} \left( \frac{\left(\frac{H}{K_H}\right)^N \cdot A_{\text{Target}}}{\left(\frac{H}{K_H}\right)^N + 1} + \frac{1}{\left(\frac{TF}{K_{TF}}\right)^N + 1} \right) - \alpha_{\text{Target}} \cdot \text{Target} \qquad (10)$$

## 3.1.2 IFFL Type 2 Equations

In Type 2, HIF-1$\alpha$ represses TF but activates the target gene, with the TF still acting as a repressor. This configuration introduces an opposite regulation on TF compared to Type 1.

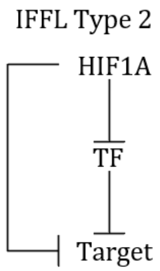


**Supplementary Figure 6. Schematic diagram of the Incoherent Feed-Forward Loop (IFFL) Type 2 motif. In this motif,** HIF1A represses both the intermediate transcription factor (TF) and the target gene. The TF also represses the target gene, resulting in an overall incoherent regulatory structure.

### 3.1.2.1 Multiplicative Dynamics

$$\frac{d\text{TF}}{dt} = \frac{\beta_{TF}}{\left(\frac{H}{K_H}\right)^N + 1} - \alpha_{\text{TF}} \cdot \text{TF} \qquad (11)$$

$$\frac{d\text{Target}}{dt} = \beta_{\text{Target}} \left( \frac{1}{\left(\left(\frac{H}{K_H}\right)^N + 1\right)\left(\left(\frac{TF}{K_{TF}}\right)^N + 1\right)} + A_{\text{Target}} \right) - \alpha_{\text{Target}} \cdot \text{Target} \qquad (12)$$

### 3.1.2.2 Additive Dynamics

$$\frac{d\mathrm{TF}}{dt} = \frac{\beta_{TF}}{\left(\frac{H}{K_H}\right)^N + 1} - \alpha_{\mathrm{TF}} \cdot \mathrm{TF} \tag{13}$$

$$\frac{d\mathrm{Target}}{dt} = \beta_{\mathrm{Target}} \left( \frac{A_{\mathrm{Target}}}{\left(\frac{H}{K_H}\right)^N + 1} + \frac{1}{\left(\frac{TF}{K_{TF}}\right)^N + 1} \right) - \alpha_{\mathrm{Target}} \cdot \mathrm{Target} \tag{14}$$

## 3.1.3 IFFL Type 3 Equations

In Type 3, HIF-1$\alpha$ activates the TF, and the TF activates the Target, while HIF-1$\alpha$ inhibits the Target. This configuration introduces competing activation and inhibition at the target level.

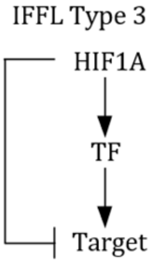


**Supplementary Figure 7. Schematic diagram of the Incoherent Feed-Forward Loop (IFFL) Type 3 motif.** In this motif, HIF1A activates an intermediate transcription factor (TF), which in turn activates the target gene. Meanwhile, HIF1A directly represses the expression of the same target gene.

## 3.1.3.1 Multiplicative Dynamics

$$\frac{d\mathrm{TF}}{dt} = \frac{\left(\frac{H}{K_H}\right)^N}{\left(\frac{H}{K_H}\right)^N + 1} \cdot \beta_{TF} - \alpha_{TF} \cdot TF \tag{15}$$

$$\frac{dTarget}{dt} = \beta_{Target} \left( \frac{\left(\frac{TF}{K_{TF}}\right)^N}{\left(\frac{TF}{K_{TF}}\right)^N + 1} + A_{Target} \right) \frac{1}{\left(1 + \left(\frac{H}{K_H}\right)^N\right)} - \alpha_{Target} \cdot Target \tag{16}$$

### 3.1.3.2 Additive Dynamics

$$\frac{d\mathrm{TF}}{dt} = \frac{\beta_{TF}}{\left(\frac{H}{K_H}\right)^N + 1} - \alpha_{\mathrm{TF}} \cdot \mathrm{TF} \qquad (17)$$

$$\frac{dTarget}{dt} = \beta_{Target} \cdot \left( \frac{\left(\frac{TF}{K_{TF}}\right)^N}{\left(\frac{TF}{K_{TF}}\right)^N + 1} + \frac{A_{Target}}{1 + \left(\frac{H}{K_H}\right)^N} \right) - \alpha_{Target} \cdot Target \qquad (18)$$

### 3.1.4. IFFL Type 4 Equations

Finally, Type 4 describes a configuration where HIF-1$\alpha$ represses TF but activates the target gene via both direct and TF-dependent pathways.

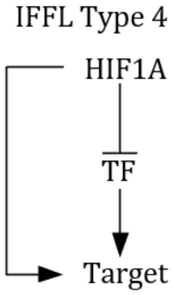


**Supplementary Figure 8. Schematic diagram of the Incoherent Feed-Forward Loop (IFFL) Type 4 motif.** In this motif, HIF1A represses the transcription factor (TF) while directly activating the target gene. The TF also activates the target gene, resulting in opposing effects of HIF1A on the target through the direct and indirect paths.

### 3.1.4.1 Multiplicative Dynamics

$$\frac{d\mathrm{TF}}{dt} = \frac{\beta_{TF}}{\left(\frac{H}{K_H}\right)^N + 1} - \alpha_{TF} \cdot TF \qquad (19)$$

$$\frac{d\mathrm{Target}}{dt} = \beta_{Target} \left( \frac{\left(\frac{H}{K_H}\right)^N}{\left(\frac{H}{K_H}\right)^N + 1} \cdot \frac{\left(\frac{TF}{K_{TF}}\right)^N}{\left(\frac{TF}{K_{TF}}\right)^N + 1} + A_{Target} \right) - \alpha_{Target} \cdot Target \qquad (20)$$

### 3.1.4.2 Additive Dynamics

$$\frac{d\mathrm{TF}}{dt} = \frac{\beta_{TF}}{\left(\frac{H}{K_H}\right)^N + 1} - \alpha_{TF} \cdot TF \tag{21}$$

$$\frac{d\mathrm{Target}}{dt} = \beta_{\mathrm{Target}} \left( \frac{\left(\frac{H}{K_H}\right)^N \cdot A_{\mathrm{Target}}}{\left(\frac{H}{K_H}\right)^N + 1} + \frac{\left(\frac{TF}{K_{TF}}\right)^N}{\left(\frac{TF}{K_{TF}}\right)^N + 1} \right) - \alpha_{\mathrm{Target}} \cdot \mathrm{Target} \tag{22}$$

## 3.2 Parameters and Estimates

### 3.2.1 Default parameters and initial conditions used in our model

The default parameters and initial conditions used in the simulations are listed in **Supplementary Table 1**. All values were either normalized or non-dimensionalized for computational simplicity. These default values were applied consistently across all simulations unless otherwise specified.

**Supplementary Table 1. Default Parameters and Initial Conditions Used in the IFFL Model**

| Parameter | Definition | Default Value |
|---|---|---|
| H | Concentration of HIF-1 | 0 |
| TF | Concentration of the transcription factor | 0 |
| Target | Concentration of the target gene product | 0 |
| $K_H$ | Dissociation constant for HIF-1 (half-maximum response) | 0.35 |
| $K_{TF}$ | Dissociation constant for TF at the target gene | 0.75 |
| $\alpha_{TF}$ | Degradation rate of TF | 1 |
| $\beta_{TF}$ | Maximum production rate of TF | 1 |
| $\alpha_{Target}$ | Degradation rate of the target gene | 1 |
| $\beta_{Target}$ | Maximum production rate of the target gene influenced by TF | 1 |
| $A_{Target}$ | Additional activation of the target gene independent of TF | 1 |
| N | Hill coefficient (cooperativity of HIF-1 binding) | 4 |

### 3.2.2 Literature-Based Estimates for Biochemical Parameters

We compiled representative biochemical parameters from published experimental studies and the BioNumbers database[6] to illustrate typical magnitudes of binding affinities(**Supplementary Table 2**), production rates(**Supplementary Table 3**), and degradation rates(**Supplementary Table 4**). While our model uses normalized, unitless parameters, these values offer biologically reasonable references that guided the selection of default settings.

**Supplementary Table 2. Representative Binding (Dissociation Constant, $K_H$,$K_{TF}$) Values**

| Property | Protein-DNA Interaction | K_d (nM) | BioNumbers ID/ References |
|---|---|---|---|
| Dissociation constant of GAGA transcription factor from DNA | GAGA transcription factor – DNA (*Drosophila*) | **5.2** nM | BNID 104594 https://pubmed.ncbi.nlm.nih.gov/17105198/ |
| First rate dissociation constant for the binding of the Egr-1 ZFD to Synthetic DNA Duplex | Egr-1 zinc-finger domain – synthetic DNA (*Human*) | ≈ **0.5** nM | BNID 104606 https://pubmed.ncbi.nlm.nih.gov/17105198/ |

**Supplementary Table 3. Representative Maximum Production Rate ($\beta_{TF}$,$\beta_{Target}$) Values**

| System / Context | Transcription Rate (mRNA/hr) | Translation Rate (proteins/mRNA/hr) | Conversion (molecules/nM) | β_total (nM/hr per gene) | BioNumbers References |
|---|---|---|---|---|---|
| Mouse NIH3T3 median gene | ~2.0 | 140 | 600 | **0.47** | BNID 106379, BNID 106382 |
| Mouse NIH3T3 highly expressed gene | ~2.0 | 1000 | 600 | **3.33** | BNID 106379, BNID 106382 |

| | | | | | |
|---|---|---|---|---|---|
| Drosophila Bicoid (embryo) | – | 120 | 600 | **0.20 (per mRNA)** | BNID 111203 |
| E. coli typical gene | – | 402 | 600 | **0.67 (per mRNA)** | BNID 111689 |
| Mammalian max observed gene (estimate) | ~3.0 | 1000 | 600 | **5** | BNID 106379, BNID 106382 |

**Supplementary Table 4. Representative Degradation rate ($\alpha_{TF}$,$\alpha_{Target}$) Values**

| **Protein / System** | **Half -life (h)** | **$\alpha$ ($h^{-1}$)** | **Degradation @ 1 nM** | **BioNumbers ID/ References** |
|---|---|---|---|---|
| d1EGFP in HEK293T (very fast decay) | 1 | 0.693 | 0.693 | https://bionumbers.hms.harvard.edu/bionumber.aspx?id=106866 https://pubmed.ncbi.nlm.nih.gov/18988847/ |
| d4EGFP in HEK293T (moderate decay) | 4 | 0.173 | 0.173 | https://bionumbers.hms.harvard.edu/bionumber.aspx?id=106866 https://pubmed.ncbi.nlm.nih.gov/18988847/ |
| EGFP in HEK293T (stable reporter) | 24 | 0.029 | 0.029 | https://bionumbers.hms.harvard.edu/bionumber.aspx?id=106866 https://pubmed.ncbi.nlm.nih.gov/18988847/ |
| Firefly luciferase in 293T cells | 2 | 0.347 | 0.347 | https://bionumbers.hms.harvard.edu/bionumber.aspx?id=101268 https://pubmed.ncbi.nlm.nih.gov/15162459/ |
| Wild-type GFP in mouse LA-9 cells | 26 | 0.027 | 0.027 | https://bionumbers.hms.harvard.edu/bionumber.aspx?id=105181 https://pubmed.ncbi.nlm.nih.gov/10611396/ |

| | | | | |
|---|---|---|---|---|
| Average human protein (H1299 cells) | 6.9 | 0.1 | 0.1 | https://bionumbers.hms.harvard.edu/bionumber.aspx?id=106875 https://pubmed.ncbi.nlm.nih.gov/21233346/ |
| Median mammalian protein (NIH3T3) | 46 | 0.015 | 0.015 | https://bionumbers.hms.harvard.edu/bionumber.aspx?id=106377 https://pubmed.ncbi.nlm.nih.gov/21593866/ |
| Median non-dividing HeLa proteome | 35.5 | 0.02 | 0.02 | https://bionumbers.hms.harvard.edu/bionumber.aspx?id=109752 https://pubmed.ncbi.nlm.nih.gov/22050367/ |

## 3.3. qRT-PCR Validation of Gene Knockdown Efficiency

We performed knockdown of **TP53** and **NOTCH1** using sgRNAs in MDA-MB-231 cells. Knockdown efficiency was confirmed by qRT-PCR, showing over 75% reduction in mRNA levels compared to scrambled controls. The validation results are shown in **Supplementary Figure 9.**

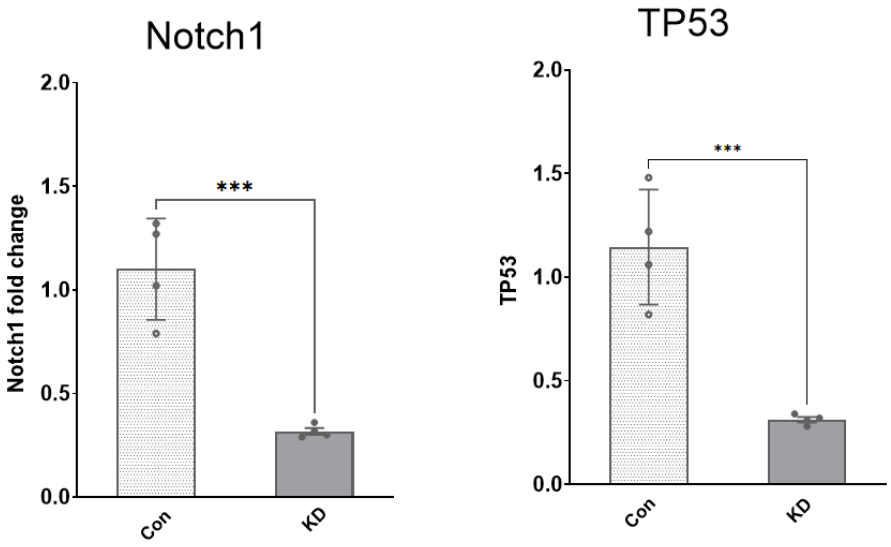


**Supplementary Figure 9. Validation of siRNA-mediated knockdown efficiency for NOTCH1 and TP53.** Expression levels were normalized to control cells. Bars represent mean ± SD (n = 4). Statistical analysis was performed using a two-tailed unpaired t-test. ***$p < 0.001$.

## Supplementary References


1 Semenza, G. L. HIF-1, O(2), and the 3 PHDs: how animal cells signal hypoxia to the nucleus. *Cell* **107**, 1-3 (2001). https://doi.org/10.1016/s0092-8674(01)00518-9

2 Wang, G. L., Jiang, B. H., Rue, E. A. & Semenza, G. L. Hypoxia-inducible factor 1 is a basic-helix-loop-helix-PAS heterodimer regulated by cellular O2 tension. *Proc Natl Acad Sci U S A* **92**, 5510-5514 (1995). https://doi.org/10.1073/pnas.92.12.5510

3 Berra, E., Roux, D., Richard, D. E. & Pouyssegur, J. Hypoxia-inducible factor-1 alpha (HIF-1 alpha) escapes O(2)-driven proteasomal degradation irrespective of its subcellular localization: nucleus or cytoplasm. *EMBO Rep* **2**, 615-620 (2001). https://doi.org/10.1093/embo-reports/kve130

4 Jewell, U. R. *et al.* Induction of HIF-1alpha in response to hypoxia is instantaneous. *FASEB J* **15**, 1312-1314 (2001).

5 Ehrismann, D. *et al.* Studies on the activity of the hypoxia-inducible-factor hydroxylases using an oxygen consumption assay. *Biochem J* **401**, 227-234 (2007). https://doi.org/10.1042/BJ20061151

6 Milo, R., Jorgensen, P., Moran, U., Weber, G. & Springer, M. BioNumbers--the database of key numbers in molecular and cell biology. *Nucleic Acids Res* **38**, D750-753 (2010). https://doi.org/10.1093/nar/gkp889
